# Pitavastatin treatment remodels the HDL subclass lipidome and proteome in hypertriglyceridemia

**DOI:** 10.1016/j.jlr.2023.100494

**Published:** 2023-12-29

**Authors:** M. John Chapman, Alexina Orsoni, Natalie A. Mellett, Anh Nguyen, Paul Robillard, Jonathan E. Shaw, Philippe Giral, Patrice Thérond, Debi Swertfeger, W. Sean Davidson, Peter J. Meikle

**Affiliations:** 1 Cardiovascular Disease Prevention Unit, Pitié-Salpetrière University Hospital, Sorbonne University and National Institute for Health and Medical Research (INSERM), Paris, France; 2 Service de Biochimie, AP–HP, Paris-Saclay University, Bicetre University Hospital, and EA 7357, Paris–Saclay University, Orsay, France; 3 Metabolomics Laboratory, Baker Heart and Diabetes Institute, Melbourne, Victoria, Australia; 4 INSERM UMR1166 and Cardiovascular Prevention Units, ICAN–Institute of CardioMetabolism and Nutrition, AP–HP, Pitie–Salpetriere University Hospital, Paris, France; 5 Department of Endocrinology, Cincinnati Children’s Hospital Medical Center, Cincinnati, OH, USA; 6 Department of Pathology and Laboratory Medicine, University of Cincinnati, Cincinnati, OH, USA; 7 Baker Department of Cardiovascular Research, Translation and Implementation, La Trobe University, Bundoora, Victoria, Australia

**Keywords:** nondiabetic hypertriglyceridemia, pitavastatin calcium, HDL subclasses, lipidomics, proteomics, metabolic remodeling

## Abstract

HDL particles vary in lipidome and proteome, which dictate their individual physicochemical properties, metabolism, and biological activities. HDL dysmetabolism in nondiabetic hypertriglyceridemia (HTG) involves subnormal HDL-cholesterol and apoAI levels. Metabolic anomalies may impact the qualitative features of both the HDL lipidome and proteome. Whether particle content of bioactive lipids and proteins may differentiate HDL subclasses (HDL2b, 2a, 3a, 3b, and 3c) in HTG is unknown. Moreover, little is known of the effect of statin treatment on the proteolipidome of hypertriglyceridemic HDL and its subclasses. Nondiabetic, obese, HTG males (n = 12) received pitavastatin calcium (4 mg/day) for 180 days in a single-phase, unblinded study. ApoB-containing lipoproteins were normalized poststatin. Individual proteolipidomes of density-defined HDL subclasses were characterized prestatin and poststatin. At baseline, dense HDL3c was distinguished by marked protein diversity and peak abundance of surface lysophospholipids, amphipathic diacylglycerol and dihydroceramide, and core cholesteryl ester and triacylglycerol, (normalized to mol phosphatidylcholine), whereas light HDL2b showed peak abundance of free cholesterol, sphingomyelin, glycosphingolipids (monohexosylceramide, dihexosylceramide, trihexosylceramide, and anionic GM3), thereby arguing for differential lipid transport and metabolism between subclasses. Poststatin, bioactive lysophospholipid (lysophosphatidylcholine, lysoalkylphosphatidylcholine, lysophosphatidylethanolamine, and lysophosphatidylinositol) cargo was preferentially depleted in HDL3c. By contrast, baseline lipidomic profiles of ceramide, dihydroceramide and related glycosphingolipids, and GM3/phosphatidylcholine were maintained across particle subclasses. All subclasses were depleted in triacylglycerol and diacylglycerol/phosphatidylcholine. The abundance of apolipoproteins CI, CII, CIV, and M diminished in the HDL proteome. Statin treatment principally impacts metabolic remodeling of the abnormal lipidome of HDL particle subclasses in nondiabetic HTG, with lesser effects on the proteome.

Hypertriglyceridemia (HTG), resulting from elevated levels of VLDL and remnants in the fasting period, is common in cardiometabolic disorders, including type 2 diabetes and the metabolic syndrome (1, 2). Evidence shows that these particles causally contribute to atherosclerotic cardiovascular disease (3, 4).

Metabolically, HTG is linked to low HDL-C, with altered HDL particles contributing to cardiovascular risk (3, 5, 6). Metabolic factors affecting the triglyceride (TG)-rich lipoprotein (TRL)-HDL axis in HTG include: (i) TG enrichment of HDL due to enhanced cholesteryl ester transfer protein (CETP)-mediated TG transfer from TRL to HDL with depletion of cholesteryl ester (CE) (3, 5), (ii) elevated hepatic lipase (HL)-mediated lipolysis of HDL TG, reducing HDL levels (6), (iii) excess small phospholipid (PL)-depleted HDL due to endothelial lipase (EL)- and HL-mediated hydrolysis of surface PL in large HDL particles, with accelerated renal clearance of HDL apoAI (6, 7), and (iv) lower capacity of HDL to acquire free cholesterol (COH) and PL from TRL during lipolysis (8). Together, such effects may impair HDL function, including antidiabetogenic, antioxidative, antiapoptotic, anti-inflammatory, vasoprotective, and vasodilatory properties (5, 9, 10, 11, 12).

The molecular composition of HDL, comprising a proteome, lipidome and minor amounts of small noncoding RNAs, lipid-soluble vitamins, steroid hormones, and more, impacts its structure, metabolism, and function (9, 12, 13, 14, 15). Aside from the dominant scaffold proteins apoAI and apoAII, a total of up to 50 additional proteins are typically detected in individual HDL isolates (14, 16). However, some 250 protein components have been identified across multiple studies involving different isolation procedures, thereby suggesting implication of HDL in cholesterol homeostasis, immunity, protease inhibition, diabetes protection, and defence against infection (16). Most of these minor proteins are present at low concentration (<1 μM) in plasma, precluding their presence on all HDL particles; their distribution thus drives particle heterogeneity (17, 18). The HDL lipidome is similarly complex, with some 300 molecular lipid species per isolate (19). Many of the minor lipids exhibit potent biological activities, including diacylglycerol (DAG), phosphatidylinositol (PI), phosphatidylserine (PS), lysophosphatidylcholine (LPC), other lysophospholipids [lysophosphatidylethanolamine (LPE), lysophosphatidylinositol (LPI)], phosphatidic acid, sphingomyelin (SM), ceramide (Cer), plasmalogen, and sphingosine-1-phosphate (S1P) (5, 12, 14, 20, 21, 22). Moreover, such lipids may be differentially distributed between HDL particle subspecies (5, 14, 20, 21, 22).

In nondiabetic and diabetic HTG states, HDL subclass patterns are altered, with decrease in large HDL2 particles and increases in small, TG-rich HDL3 (3, 5, 12). Considering HDL as a whole, lipidomic shifts involve elevation in LPCs, triacylglycerols (TAGs), and DAGs, and lower abundance of phosphatidylcholine (PC)-based, PE-based plasmalogens, S1P, SMs, and CE (as % wt) (5, 10, 12, 23, 24, 25, 26). Proteome changes involve reduced apoAI, apoAIV, apoD, apoE, apoF, clusterin, and apoM contents, but increased serum amyloid A, apoCII, apoCIII, apoE, and fibrinogen (5, 10, 12, 27). There is however a paucity of data on the lipidomic and proteomic profiles of HDL subclasses, that is, HDL2b, 2a, 3a, 3b, and 3c, in nondiabetic HTG.

Statins, or HMGCoA reductase inhibitors, are first line treatment for reduction in cardiovascular risk in dyslipidemias involving HTG and low HDL-C, and target lowering of atherogenic apoB-containing lipoproteins, including VLDL, remnants, and LDL (28, 29). Statin therapy equally induces minor increase in HDL-C and apoAI levels (<15%), with dependency on the specific statin, dose, duration of exposure, baseline level, and patient characteristics (29, 30). Furthermore, statins impact HDL remodeling via effects on lipases (HL and EL), lipid transfer proteins (CETP and PL transfer protein, PLTP) and lipid esterifying enzymes (LCAT) (31, 32, 33). However, the effect of statin treatment on the lipidomic and proteomic profiles of HDL subclasses is not well understood. These key questions were evaluated in nondiabetic, hypertriglyceridemic subjects after treatment with pitavastatin calcium (4 mg/day) for 180 days (the CAPITAIN study; Chronic and Acute effects of PITAvastatIN on monocyte phenotype, endothelial function and HDL atheroprotective function in patients with metabolic syndrome; ClinicalTrials.gov: NCT01595828) study (33, 34, 35). Here, we defined the differential distribution of both lipids and proteins among the five major HDL subclasses in HTG and evaluated the effect of prolonged statin treatment on both the subclass lipidome and proteome in the context of the normalization of the atherogenic profile of atherogenic apoB-containing lipoproteins. Focus has been attributed to polar proinflammatory lipids, to lipids with potential action as second messengers, and to core lipids whose metabolism is primarily regulated by mechanisms of lipolysis and of lipid transfer between lipoprotein particles.

## Materials and Methods

### Dyslipidemic subjects

Key phenotypic features of the dyslipidemic subjects, the clinical protocol, inclusion and exclusion criteria, dietary counseling, and compliance have been detailed previously and are summarized in Supplemental material (33, 34, 35); essential features are summarised below and in supplemental Fig. S1. The monocentric CAPITAIN Study (ClinicalTrials.gov, #NCT01595828) recruited healthy, hypertriglyceridemic, hypercholesterolemic, obese Caucasian male subjects (n = 12; mean age 50 ± 3 years) (LDL-C, 130–190 mg/dl; 3.4–4.9 mmol/L; Lp(a) < 10 mg/dl), with HDL-C levels significantly inferior to those of the control group (Table 1) (34, 35, 36, 37, 38, 39). The elevated BMI at baseline was unchanged poststatin (31.7 ± 0.5 and 31.8 ± 0.7 kg/m^2^, respectively) (33). As noted earlier, the presence of obesity, HTG, and controlled hypertension qualified these subjects as presenting the metabolic syndrome (33); in addition, the baseline homeostasis model of insulin resistance score of 2.7 ± 1.7 placed them at the threshold for insulin resistance and was stable over the course of the study (2.2 ± 0.3 at 180 days; NS) (40). The protocol was approved by the Ethics Committee of the Pitie-Salpetriere University Hospital; the volunteers gave written informed consent. The study was performed in accordance with the ethical principles set forth in the Declaration of Helsinki. Subjects received pitavastatin calcium (dose 4 mg/day) for 180 days. Pitavastatin calcium was selected in view of evidence-based neutrality on glucose homeostasis (http://www.kowapharmaceuticals.eu/assets/dl/Livazo-SmPC-28-11- 17.pdf) (41). Given the polygenic nature of HTG, subjects acted as their own controls with respect to the effects of statin treatment in order to limit potential confounding between separate treatment and placebo groups due to (i) variation in baseline phenotype, (response to statins is phenotype-dependent) (42) and (ii) variation in genetic background and thus potential variability in pharmacogenomic response to statin therapy (43). This aspect is further discussed under « Limitations » in the Discussion section.

**Table 1:** Plasma levels of lipids, lipoprotein lipids, lipoprotein mass, apolipoproteins and biomarkers in hypertriglyceridemic male subjects at baseline (D0), the effect of treatment with Pitavastatin calcium (4 mg/day; D180) for 180 days, and comparison with a healthy, normolipidemic control group

Parameter	HTG Subjects (n = 12)			Control Subjects
	Baseline D0	D180	% Change (D180 vs. D0)	
Total cholesterol (mg/dl)	232.2 ± 17.6	161.7 ± 5.7∗∗∗	−30%	171.4 ± 8.0^§§^^a^
Triglycerides (mg/dl)	215.9 ± 16.0	127.7 ± 8.1∗∗∗	−41%	75.3 ± 11.1^§§§,###^^a^
LDL-cholesterol (mg/dl)	153.0 ± 6.2	96.1 ± 5.8∗∗∗	−37%	100.4 ± 6.4^§§§^^a^
ApoB (mg/dl)	102.0 ± 4.2	72.8 ± 5.1∗∗∗	−29%	80.3 ± 12.6^a^
Lp(a)^a^ (mg/dl)	8.8 (0.5–24.9)	8.5 (0.9–32.2)	−3%	<10.0^a^
Non-HDL-C (mg/dl)	185.9 ± 15.8	113.5 ± 4.5∗∗∗	−39%	115.0 ± 8.5^§§^
RLP-C (mg/dl)	39.1 ± 12.9	17.5 ± 4.1	−55%	14.6 ± 3.8^§,^∗
HDL-cholesterol (mg/dl)	46.3 ± 2.8	48.2 ± 3.6	+4%	56.4 ± 3.0^§^^a^
Total HDL mass (mg/dl)	201.0 ± 3.1	200.6 ± 3.9	0	261.2 ± 4.8^§§§,###^^a^
HDL2 mass (mg/dl)	103.7 ± 4.8	96.0 ± 8.9	−7%	145.1 ± 5.6^§,#^^a^
HDL3 mass (mg/dl)	97.3 ± 4.3	104.6 ± 5.8	+8%	116.1 ± 3.2^§,#^^a^
HDL2b mass (mg/dl)	39.6 ± 3.7	35.2 ± 4.1	−11%	71.4 ± 6.9^§§§,###^^a^
HDL2a mass (mg/dl)	64.1 ± 3.3	60.8 ± 5.5	−5%	73.7 ± 5.1^§,#^^a^
HDL3a mass (mg/dl)	60.5 ± 2.7	64.9 ± 3.8	+7%	67.8 ± 5.6^a^
HDL3b mass (mg/dl)	26.0 ± 1.3	28.5 ± 1.5	+10%	31.2 ± 3.2^a^
HDL3c mass (mg/dl)	10.7 ± 0.8	11.1 ± 0.8	+4%	17.1 ± 1.9^§§,##^^a^
Pre-β1-HDL (mg/dl)	6.9 ± 1.0	6.0 ± 0.9	−13%	2.3 ± 0.2^§§§,###^^b^
LpAI (mg/dl)	28.1 ± 2.1	27.8 ± 3.3	−1%	47.0 ± 0.8^§§§,###^^e^
LpAI:AII (mg/dl)	72.2 ± 3.4	78.7 ± 3.4∗	+9%	99 ± 0.9^§§§,###^^e^
ApoAI (mg/dl)	100.3 ± 4.8	106.6 ± 5.7	+6%	147.1 ± 4.2^§§§,###^^a^
ApoAII (mg/dl)	24 ± 1.0	26.1 ± 1.6	+9%	35.2 ± 0.1^§§§,###^^c^
ApoAI/ApoAII ratio	4.2 ± 0.2	4.1 ± 0.2	−2%	4.2 ± 0.1
ApoE (mg/dl)	4.3 ± 0.2	3.3 ± 0.1∗∗∗	−24%	4.1 ± 0.7^b^
ApoCII (mg/dl)	16.7 ± 2.7	6.5 ± 1.6∗∗	−61%	4.6 ± 0.4^§§§^^d^
ApoCIII (mg/dl)	10.3 ± 1.2	7.8 ± 0.8	−25%	9.9 ± 0.5^#^^d^
Sphingosine-1-phosphate (nmol/L)	187.2 ± 32.6	155.6 ± 37.8	−17%	ND
ApoM (mg/dl)	0.25 ± 0.01	0.22 ± 0.01	−10%	ND
SAA (mg/L)	26.5 ± 3	19.3 ± 2∗	−27%	ND
oxLDL (μg/dl)	84.0 ± 4.8	56.4 ± 3.6∗	−34%	ND
Lp-PLA2 mass (ng/ml)	223 ± 17	205.2 ± 15∗∗∗	−8%	ND
Lp-PLA2 activity (UI/L)	234 ± 11	192 ±12∗∗∗	−18%	ND
Paraoxonase (mU/μl)	28.1 ± 0.01	34.4 ± 0.01∗∗	+24%	ND
hsCRP (mg/L)	1.6 ± 0.2	1.6 ±0.2	0	ND
CETP mass (μg/ml)	2.4 ± 0.1	2.0 ± 0.1∗∗	−18%	ND
CETP activity (pmol/ml/min)	50.3 ± 3.0	42.3 ±2.0∗∗	−16%	ND
LCAT activity (%)	67.1 ± 0.5	68.4 ± 0.6∗∗	+2%	ND

CETP, CETP, cholesteryl ester transfer protein; ND, not determined.

Values in HTG subjects are expressed as means ± SEM (n = 12 unless stated otherwise). ND, not determined. Due to an asymmetric distribution, Lp(a) levels are expressed as median (minimum-maximum). ∗∗∗*P* < 0.001, ∗∗0.001 < *P* < 0.01, and ∗0.01 < *P* < 0.05 for values at D0 versus D180; ^§§§^*P* < 0.001, ^§§^0.001 < *P*<0.01, and ^§^0.01 < *P* < 0.05 for values at D0 versus Control subjects; ^###^*P* < 0.001, ^##^0.001 < *P* < 0.01, and ^#^0.01 < *P* < 0.05 for values at D180 versus Control group. Details of assay methods are provided in the text. Non-HDLC (as TC-HDLC) and ApoAI/ApoAII ratio were calculated with GraphPadPrism 8.4.2 using baseline-corrected function with HDL-C and ApoAII as baseline values (34). Remnant lipoprotein-cholesterol (RLP-C) was calculated as previous (34). Statistical analyses for D0 versus D180 data were performed as paired t-tests (for parameters with Gaussian distribution) or Wilcoxon’s test (for parameters without Gaussian distribution).

Data for the effect of pitavastatin treatment on several baseline parameters in plasma (total cholesterol, triglycerides, LDL-C, apoB, Lp(a), non-HDL-C, RLP-C, HDL-C, chemical masses of total HDL and HDL subclasses, pre-β1-HDL, LpAI and LpAI:AII, apoAI and apoAII, apoE, apoCII, apoCIII, CETP mass and activity and LCAT activity were extracted from reference (34). Data for the effect of statin treatment on baseline levels of oxLDL, paraoxonase activity, LpPLA2 mass and activity, and SAA levels were reported earlier (35), as were data for apoM and S1P levels (26).

a Data in control subjects (refs (34, 35), respectively) (n = 10).

b Data in control subjects (n = 25 for pre-β1-HDL; n = 10 for ApoE, respectively) (36).

c Data in control subjects (n = 1635) (37).

d Data in control subjects (n = 25) (38).

e Data in control subjects (n = 233) (39).

### Healthy normolipidemic subjects

For comparative purposes, plasma samples from overnight-fasted, healthy, age-matched, nonobese, healthy normolipidemic male subjects (n = 10) were selected from the Australian diabetes, obesity, and lifestyle study (AusDiab) as indicated earlier (33, 35). Written informed consent was given by each subject after the purpose and nature of the investigation had been explained. These subjects were consuming a Western-type diet commensurate with their BMI (mean 23.1 ± 2.5 kg/m^2^) were matched for age (49 ± 11 years) and were neither hypertensive (systolic blood pressure, 119 ± 10 mmHg) nor hyperglycemic (fasting blood glucose, 5 ± 0.7 mmol/L); none displayed a history of cardiovascular disease or type 2 diabetes.

## Experimental Procedures

All experimental procedures were approved by the review board of the medical faculty of the Pitie-Salpetriere University Hospital, Paris, France.

### Blood samples

Blood samples were collected after overnight fasting before initiation of statin treatment (baseline, D0) and at 180 days (D180) within 24 h after the final intake of drug as detailed earlier. Blood samples were withdrawn in the Clinical Unit by venipuncture from the antecubital vein into precooled (4°C) sterile, evacuated tubes in the presence or absence (for serum isolation) of EDTA (final concentration 1 mg/ml) at pretreatment baseline (D0) and posttreatment (D180) time points (17, 21, 22, 31, 33, 34, 35). Plasma or serum was separated from blood cells by low-speed centrifugation at 1,700 *g* for 20 min at 4°C; sucrose (final concentration 0.06%) was added to cryoprotect lipoproteins and plasma or serum aliquoted into sample tubes purged with nitrogen within 2 h of blood collection. After freezing in liquid nitrogen, samples were stored at −80°C under nitrogen until analysis; samples were thawed once and analyzed directly. Earlier studies have documented the absence of lipid- or protein-derived oxidation products in the component lipoproteins of such samples (44).

### Lipoprotein preparation

Preparative fractionation of HDL subclasses from EDTA plasmas at D0 and D180 was performed by single step, isopycnic density gradient ultracentrifugation in a Beckman SW41 Ti rotor at 40,000 rpm for 44 h in a Beckman XL70 ultracentrifuge at 15°C (45). Five major subclasses of HDL were isolated based on their hydrated densities, that is, large light HDL2b (d 1.063–1.090 g/ml) and HDL2a (d 1.090–1.120 g/ml), and small dense HDL3a (d 1.120–1.150 g/ml), HDL3b (d 1.150–1.180 g/ml), and HDL3c (d 1.180–1.210 g/ml). Recoveries of total lipoprotein lipid, based on total plasma lipid content, were essentially complete for triglyceride and total cholesterol (≈98% and ≈ 97% respectively). Those of PLs were lower and more variable (range 84%–93%), possibly reflecting minor variation in the fractionation of HDL3c at its lower density limit (d 1.179 g/ml) where it is juxtaposed with PL-rich very high-density lipoproteins (45). Consistent with earlier data, recovery of total cholesterol within HDL subclasses consistently exceeded 95% (31). Further methodological details for this procedure can be found under “Preparative methods: Density gradient isolation of HDL subclasses” in Supplementary Material. Finally, the structural, physicochemical, and functional properties of native HDL particle subclasses isolated by isopycnic density gradient ultracentrifugation have been extensively documented in both healthy normolipidemic and dyslipidemic subjects (17, 21, 22, 31, 34, 35, 36, 44, 45).

For comparative purposes, the total HDL (d 1.063–1.21 g/ml) fraction and albumin-rich, very high–density HDL d>1.21 g/ml fractions were isolated by a four-step flotation procedure from the plasmas of hypertriglyceridemic subjects at D0 and D180 time points, and from plasmas of the control group, by sequential flotational ultracentrifugation (45).

### Analytical methods

Methods for determination of (i) lipid and apolipoprotein profile, (ii) the % weight chemical composition and mass of HDL subfractions, (iii) LpAI and LpAI:AII particle concentrations, (iv) plasma pre-β1-HDL levels, (v) plasma LCAT activity, (vi) plasma CETP mass and activity, (vii) plasma lipoprotein-associated phospholipase A2 mass and activity, (viii) serum amyloid (SAA) levels, (ix) serum paraoxonase activity, and (x) plasma oxidized LDL levels, at both D0 and D180 time points, have been described elsewhere (34, 44, 45).

### Lipid extraction

The order of HDL subclass samples and of total HDL (d 1.063–1.21 g/ml) from the intervention and control groups was randomized prior to lipid extraction and analysis. Total lipid extraction from a 10 μl aliquot of each subfraction was performed by a single-phase chloroform:methanol (2:1) extraction, as described previously (33, 35). Samples were analyzed in triplicate and the average values taken for subsequent statistical analyses.

### Lipidomic analyses and data expression

Analyses of the lipidomic profiles of HDL subclasses and the total HDL fraction at D0 and D180 timepoints in the CAPITAIN cohort and in the total HDL fraction in the control group were conducted by liquid chromatography followed by ESI-LC-MS) as described earlier (33, 35). For details of the analysis and quantitation of lipid species by LC-MS (supplemental Table S1), see Supplementary material.

The lipidomic data for each of 23 lipid classes in each HDL subclass and in the total HDL fraction were expressed: (i) as plasma mass concentration of each lipid class at baseline (D0) and after statin treatment (D180), (ii) on a per particle basis, as molar concentrations of each lipid class in individual HDL subclasses relative to the dominant surface lipid, that is PC, at D0 and D180, and (iii) as molar concentrations of each lipid class relative to that of apoAI, the most abundant protein component of all HDL subclasses. For each molar ratio of a specific lipid class/moles PC or per moles apoAI, the value at baseline was compared with the corresponding poststatin value in the same HDL subclass using a repeated measures ANOVA (corrected for multiple comparisons by the Benjamini–Hochberg method) with students *t* test post hoc analysis corrected for multiple comparisons by the Dunn-Sidak method. Comparison of lipid class/PC and lipid class/apoAI values between HDL subclasses was performed separately by Bonferroni’s posttest to a two-way repeated measure ANOVA.

### Proteomic analyses and data expression

Samples of all five HDL subclasses and of the total HDL fraction (150 μg protein) corresponding to both T0 and T180 time points were dialyzed into 50 mM ammonium bicarbonate buffer and total protein content determined by the Markwell modified Lowry protein assay (46); dialyzed samples were subsequently freeze-dried in borosilicate tubes. Following delipidation by chloroform/methanol extraction, each protein pellet was resuspended in 20% methanol/80% ammonium bicarbonate (90 μl). Solubilized protein was reduced in 10 mM dithiothreitol (30 min), followed by carboxymethylation in 40 mM iodoacetamide for 30 min. Delipidated protein (50 μg) was treated overnight at 37°C with 5% sequencing grade trypsin, followed by a 2 h incubation with an additional 2.5% trypsin. Each protein sample (30 pmol, determined using an average molecular weight for HDL proteins of 25,000) was injected onto a C18 capillary reversed phase column (Vydac, 500 μm X 15 cm) on a capillary HPLC (Agilent 1100) and eluted on an acetonitrile gradient of 0–40% with 0.1%TFA for 120 min at 6.0 μl/min. The eluting peaks were subjected to ESI-MS detection on a Sciex/Applied Biosystems QSTAR XL mass spectrometer equipped with an electrospray ionizer and a quadropole-Tof dual analyzer in the range 300–1800 *m/z*. Automated MS/MS sequencing was carried out between 100 and 2000 *m/z* in Q2 pulsing mode. The instrument was externally calibrated using a CsI and [Glu1]-Fibrinopeptide B (Sigma, St. Louis, MO) prior to each set of runs.

For a top-line qualitative analysis, the MS/MS data was used to identify proteins using Mascot (version 2.2.07) and X1 Tandem (2010.12.01.1). The data was collected in Scaffold (version 4.3.3) with peptide and protein thresholds set at 99% with two peptides required for identification. Quantitation was performed using MaxQuant (ver. 1.6.8) with unmodified.wiff files from the mass spectrometer allowing match between runs, one peptide per protein, and a 5% false discovery rate (47). The human SwissProt FastA database (2019; 73,928 total proteins) was used with fixed carbamidomethyl modification, variable methionine oxidations, and acetylation of the N terminus allowed. Untargeted label-free quantitation (LFQ) intensity values were analyzed by LFQAnalyst to identify differences in levels of individual HDL proteins (with respect to the total proteins in the sample) between prestain and poststatin treatment for: i) individual HDL subclasses and ii) summed as total HDL for each subject (48). The statistical analysis used paired *t* tests adjusted for multiple comparisons using Benjamin–Hochberg false discovery rate correction and Perseus type imputations. In some cases, involving lower abundance proteins that could not be effectively analyzed by MaxQuant, an MS1 full-scan filtering analysis was performed from data-dependent acquisition experiments using Skyline (49). Trends with respect to statin treatment were detected using a two-tailed *t* test without accounting for multiple comparisons. Pearson moment-product correlation coefficients were calculated to evaluate relationships between variables. Correlations among proteins across the HDL density subclasses were additionally analyzed using the organic algorithm of the Cytoscape software package (50). Data are shown as means ± 1 sample SD.

### Statistical analyses

Given the limited size of our male HTG cohort (n = 12), all statistical analyses were conducted initially using parametric tests, and confirmed based on a nonparametric test. Further details of statistical analyses and corrections for multiple comparisons are provided in Supplementary materials.

## Results

### Comparison of baseline plasma phenotype in HTG subjects with controls and effect of pitavastatin

HTG subjects displayed an atherogenic lipid profile with significantly elevated plasma levels of TG, LDL-, non-HDL- and remnant lipoprotein (RLP)-cholesterol, and apoB at baseline (Table 1) (33, 34, 35). By contrast, baseline levels of HDL-C, total HDL mass, total HDL2 and HDL3 mass, apoAI, apoAII, and both LpAI and LpAI:AII were subnormal; equally, baseline concentrations of HDL2b, 2a, and 3c subclasses (as total chemical mass) were markedly lower than corresponding levels in controls (Table 1). Plasma apoCII levels were supranormal (3-fold) in the HTG group, whereas apoCIII and apoE concentrations were comparable to those in controls (Table 1).

Pitavastatin treatment for 180 days substantially normalized TG levels and atherogenic apoB-containing lipoproteins (Table 1) (33, 34, 35). However, levels of HDL-C, total HDL mass, total HDL2 and HDL3 mass, HDL2b, 2a, 3a, 3b and 3c mass, and apoAI and apoAII remained subnormal, showing minor changes poststatin (Table 1 and supplemental Table S2). Content of LpAI in HDL3a was reduced at the expense of elevation in LpAI:AII in HDL2a and 3a, suggesting statin-driven redistribution of apoAI and apoAII between subclasses. Furthermore, the low baseline neutral lipid core ratio of CE/TG across HDL subclasses (range from 2.2/1 to 3.4/1) trended toward corresponding control values poststatin (poststatin, range from 3.7/1 to 5.6/1, p for trend <0.05 vs. baseline; controls, range from 4.3/1 to 6.3/1) (supplemental Table S2) (34). A pronounced fall in apoCII levels (−61%) with normalization was observed poststatin, consistent with enhanced lipolysis and clearance of VLDL and intermediate-density lipoprotein (IDL) (d < 1.019 g/ml; −29% mass) (Table 1) (34).

In summary, the atherogenic apoB-containing lipoprotein profile in HTG, including RLP-cholesterol, was largely normalized poststatin. In contrast, metrics of plasma HDL remained subnormal.

### Total HDL: comparison of baseline lipid class concentrations in the HTG group with controls and with pitavastatin treatment

Concentrations of 23 lipid classes in total HDL (d 1.063–1.21 g/ml) pre-and poststatin determined by LC-MS were normalized to apoAI and compared to values in control subjects (expressed as mean % difference) (Table 2). Baseline comparison of the HTG and control groups confirmed markedly reduced abundance of multiple lipid classes in HDL in the HTG group. Indeed, deficits of ≈ 40% or more were seen in COH, SM, alkylphosphatidylcholine (PC(O)), phosphatidylcholine plasmalogen (PC(P)), LPC, lysoalkylphosphatidylcholine (LPC(O)), alkylphosphatidylethanolamine (PE(O)), phosphatidylethanolamine plasmalogen (PE(P)), LPE, PI, LPI, PS, and Cer, monohexosylceramide (MHC), dihexosylceramide (DHC), trihexosylceramide (THC), and GM3 ganglioside (GM3). By contrast, enrichment in both DAG and TAG was observed (15.6%–27.4%; nonsignificant, NS) in HTG HDL versus corresponding values in controls.

**Table 2:** Comparison of lipid/apoAI molar ratios at baseline (D0) in the total HDL fraction (d 1.063–1.21 g/ml) in HTG subjects with healthy controls and the effect of pitavastatin treatment (D180) on baseline values in the HTG group

Lipid Class	Baseline (D0) versus Control		Poststatin (D180) versus Baseline (D0)	
	Mean % Difference^a^	*P*-value^b^	Mean % Difference^c^	*P*-value^b^
Dihydroceramide	−36.6	**2.54E-05**	26.6	**3.12E-02**
Ceramide	−49.7	**5.35E-06**	40.3	**7.71E-03**
Monohexocylceramide	−52.7	**8.25E-06**	37.5	**1.32E-02**
Dihexosylceramide	−52.3	**1.43E-05**	33.2	**2.03E-02**
Trihexosylceramide	−55.5	**5.35E-06**	29.3	**6.76E-03**
GM3 ganglioside	−55.1	**1.37E-08**	39.4	**6.76E-03**
Sphingomyelin	−47.5	**3.76E-06**	49.2	**6.76E-03**
Phosphatidylcholine	−37.3	**3.94E-06**	30.9	**1.95E-02**
Alkylphosphatidylcholine	−45.8	**2.57E-04**	43.0	**6.76E-03**
Phosphatidylcholine plasmalogen	−54.7	**1.43E-05**	40.1	**6.76E-03**
Lysophosphatidylcholine	−50.0	**3.97E-03**	12.9	6.49E-01
Lysoalkylphosphatidylcholine	−49.0	**1.11E-04**	13.9	4.62E-01
Phosphatidylethanolamine	−28.9	1.51E-01	24.1	1.30E-01
Alkylphosphatidylethanolamine	−58.2	**1.88E-04**	62.9	6.69E-02
Phosphatidylethanolamine plasmalogen	−59.4	**4.63E-05**	51.2	**7.71E-03**
Lysophosphatidylethanolamine	−42.7	**1.37E-03**	11.6	5.47E-01
Phosphatidylinositol	−40.2	**2.11E-05**	20.0	2.75E-01
Lysophosphatidylinositol	−73.7	**2.15E-03**	109.2	7.59E-02
Phosphatidylserine	−52.0	**6.50E-04**	12.4	7.63E-01
Free cholesterol	−48.4	**2.58E-06**	28.6	**1.32E-02**
Cholesteryl ester	−27.1	**1.76E-04**	18.9	**3.31E-02**
Diacylglycerol	27.4	1.10E-01	−4.3	7.63E-01
Triacylglycerol	15.6	3.83E-01	−4.6	7.63E-01

CE, cholesteryl ester; Cer, ceramide; COH, free cholesterol; DAG, diacylglycerol; dhCer, dihydroceramide; DHC, dihexosylceramide; GM, GM3 ganglioside; LPI, lysophosphatidylinositol; MHC, monohexocylceramide; PC, phosphatidylcholine; PC(O), alkylphosphatidylcholine; PE(P), phosphatidylethanolamine plasmalogen; PI, phosphatidylinositol; PS, phosphatidylserine; SM, sphingomyelin; TAG, triacylglycerol; THC, trihexosylceramide.

a Mean percentage difference between baseline (D0) values in the HTG group (n = 12) taking Healthy Controls (n = 10) as reference.

b Significance determined by *t* test; *P*-values were corrected for multiple comparisons by the method of Benjamini–Hochberg; bold indicates corrected *P*-values <0.05.

c Mean percentage difference, taking Baseline (D0) in the HTG group as reference.

Statin treatment (D180) exerted a marked trend to increase in lipid abundance in (total) HDL (normalized to apoAI), except for DAG and TAG, which trended lower (NS) (Table 2). Poststatin increments in particle content of COH, CE, dihydroceramide (dhCer), Cer, MHC, DHC, THC, GM3, SM, PC, PC(P), and PE(P) ranged from +18.9 to +51.2% (range of *P* values, < 0.03 to < 0.007) (Table 2). Overall, pitavastatin treatment induced a marked trend toward increase in (total) HDL particle lipid content in HTG subjects.

### HDL subclasses: baseline lipidomic profiles in HTG subject normalized to moles PC or moles apoAI and effect of pitavastatin treatment

**Fig. 1:**
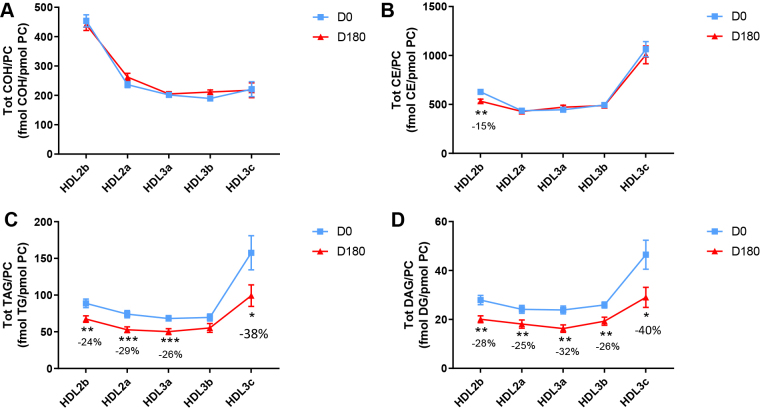

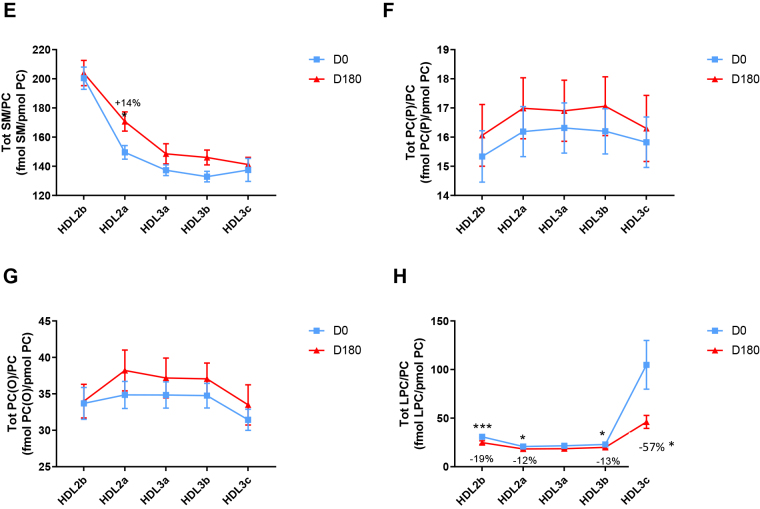

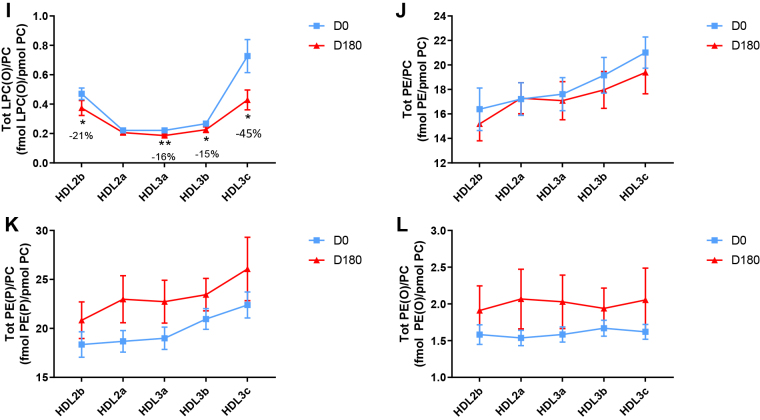

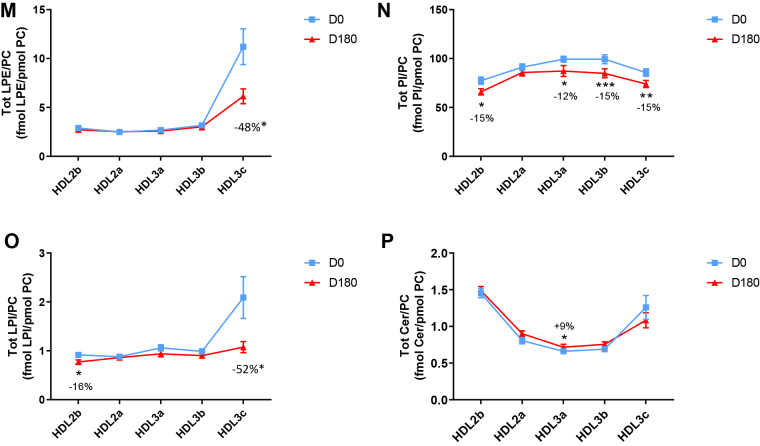

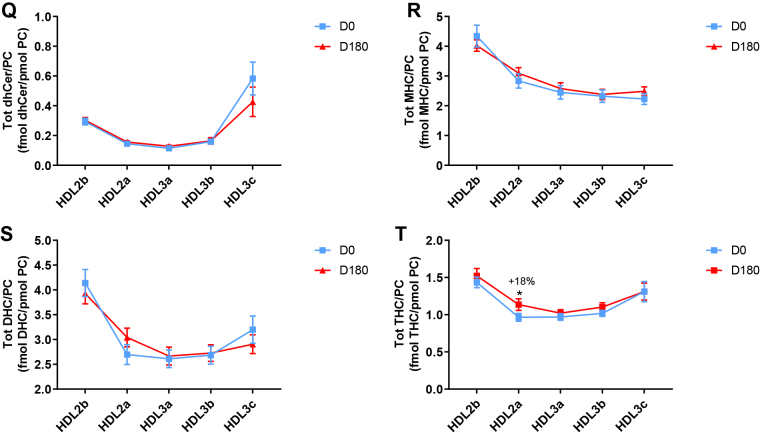

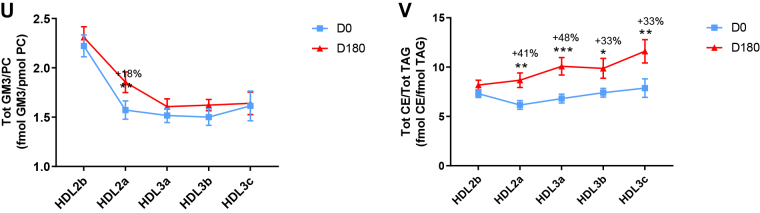
The effect of pitavastatin calcium treatment (4 mg/day) for 180 days (D180) on baseline plasma concentrations of lipid classes normalized to moles PC in HDL2b, HDL2a, HDL3a, HDL3b, and HDL3c subclasses in hypertriglyceridemic subjects. Data are expressed as means ± SEM (n = 12) in fmol of each lipid class/pmol PC. Percent change (%) was calculated relative to baseline values (D0). ∗∗∗*P* < 0.001; ∗∗0.001 < *P* < 0.01; and ∗0.01 < *P* < 0.05 versus D0. Hydrated density ranges of HDL subclasses are: HDL2b = 1.063–1.091 g/ml, HDL2a = 1.091–1.110 g/ml, HDL3a = 1.110–1.133 g/ml, HDL3b = 1.133–1.156 g/ml, and HDL3c = 1.156–1.179 g/ml. Panels A to V: A: COH, free cholesterol; B: CE, cholesteryl ester; C: TAG, triacylglycerol; D: DAG, diacylglycerol; E: SM, sphingomyelin; F: PC(P), alkenylphosphatidylcholine (plasmalogen); G: PC(O), alkylphosphatidylcholine; H: LPC, lysophosphatidylcholine; I: LPC(O), lysoalkylphosphatidylcholine; J: PE, phosphatidylethanolamine; K: PE(P), alkenylphosphatidylethanolamine (plasmalogen); L: PE(O), alkylphosphatidylethanolamine; M: LPE, lysophosphatidylethanolamine; N: PI, phosphatidylinositol; O: LPI, lysophosphatidylinositol; P: Cer, ceramide; Q, dhCer, dihydroceramide; R: MHC, monohexosylceramide; S: DHC, dihexosylceramide; T: THC, trihexosylceramide; U: GM3, monosialodihexosylganglioside; V: CE/TAG ratio.

**Fig. 2:**
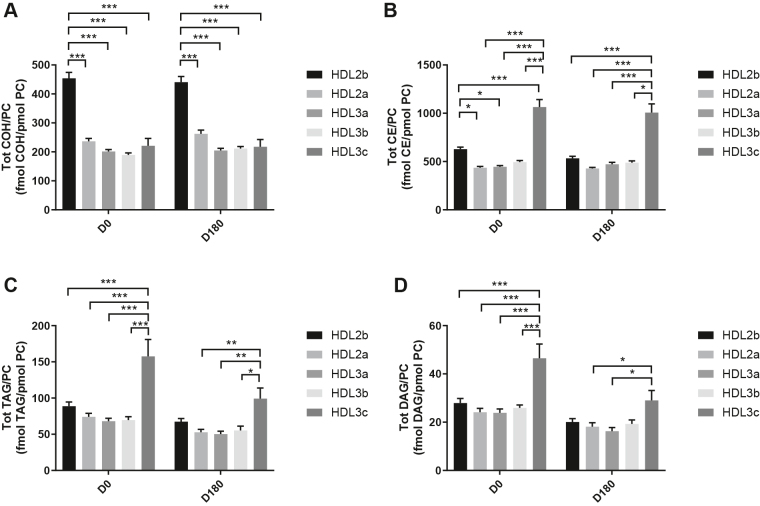

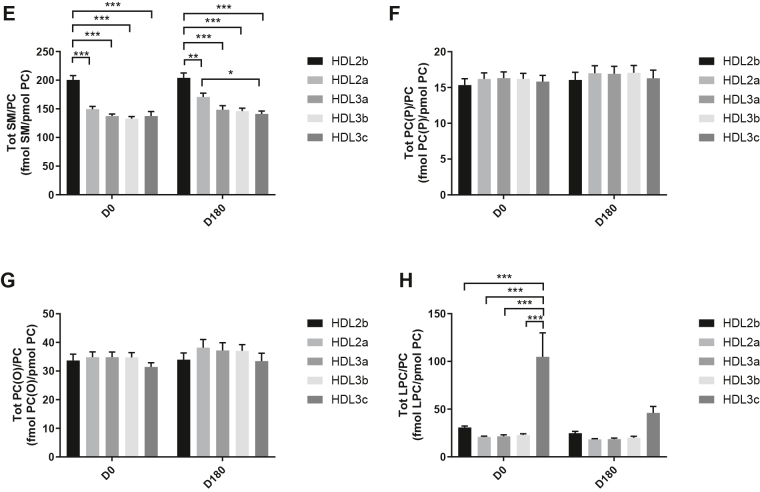

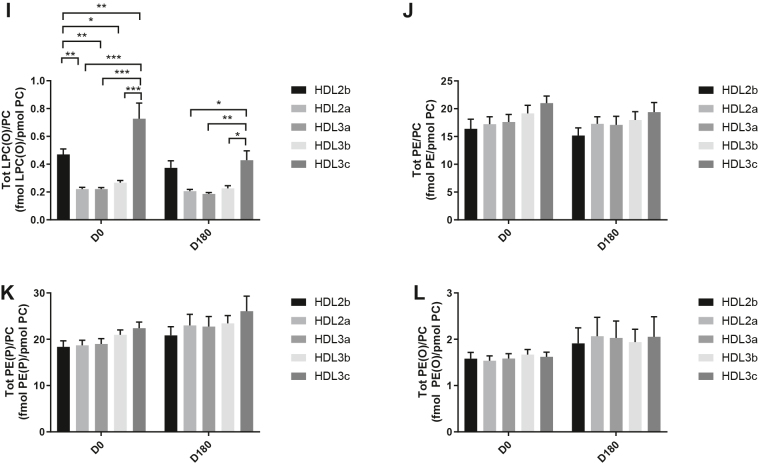

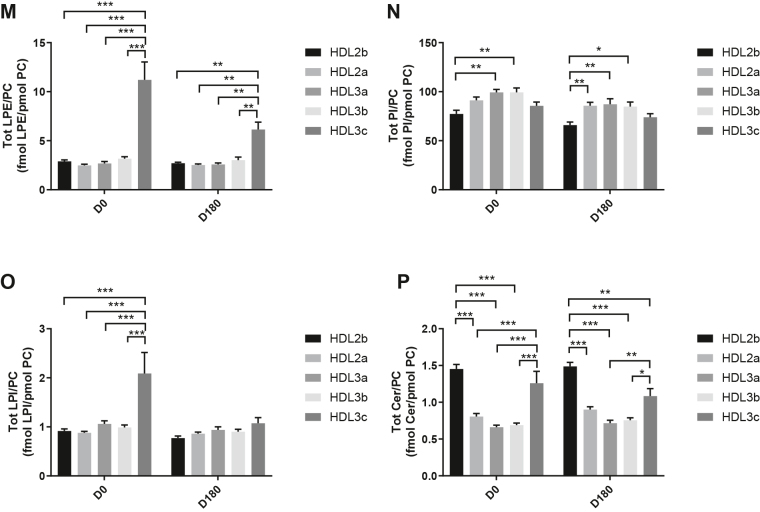

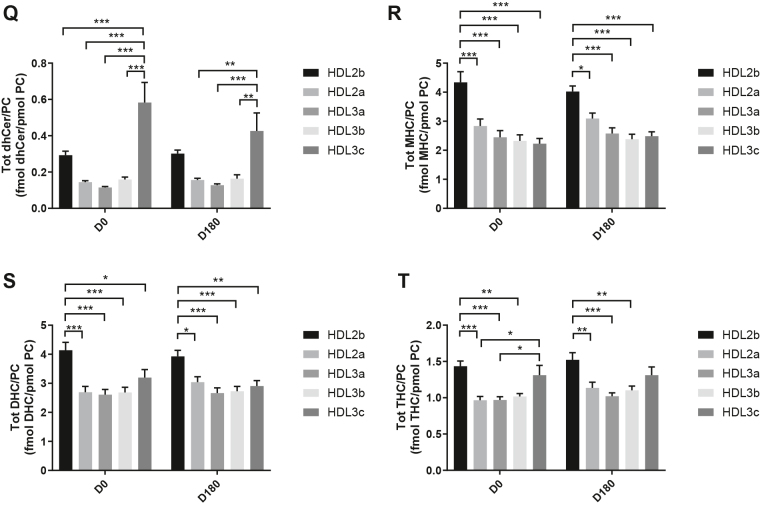

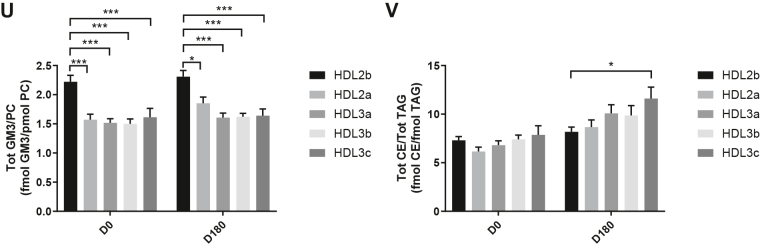
Statistical analyses of differences between plasma concentrations of lipid classes normalized to PC in HDL2b, HDL2a, HDL3a, HDL3b and HDL3c at baseline (D0) (at left) and following pitavastatin treatment (4 mg/day) for 180 days (D180) (at right) in hypertriglyceridemic subjects. Data are expressed as means ± SEM (n=12) in pmol of each lipid class/pmol PC. Density ranges: HDL2b = 1.063–1.091 g/mL, HDL2a = 1.091–1.110 g/mL, HDL3a = 1.110–1.133 g/mL, HDL3b = 1.133–1.156 g/mL, and HDL3c = 1.156–1.179 g/mL. ∗∗∗*P* < 0.001; ∗∗0.001 < *P* < 0.01; and ∗0.01 < *P* < 0.05 vs D0. Panels A to V: A – COH: free cholesterol; B – CE: cholesteryl ester; C – TAG: triacylglycerol; D – DAG: diacylglycerol; E – SM: sphingomyelin; F – PC(P): alkenylphosphatidylcholine (plasmalogen); G – PC(O): alkylphosphatidylcholine; H – LPC: lysophosphatidylcholine; I – LPC(O): lysoalkylphosphatidylcholine; J – PE: phosphatidylethanolamine; K – PE(P): alkenylphosphatidylethanolamine (plasmalogen); L – PE(O): alkylphosphatidylethanolamine; M – LPE: lysophosphatidylethanolamine; N – PI: phosphatidylinositol; O – LPI: lysophosphatidylinositol; P – Cer: ceramide; Q – dhCer: dihydroceramide; R – MHC: monohexosylceramide; S – DHC: dihexosylceramide; T – THC: trihexosylceramide; U – GM3: monosialodihexosylganglioside; V – CE/TAG ratio.

HDL2b, 2a, and 3a predominated as major transporters of all 23 lipid classes both at baseline and poststatin (for further details, see Supplemental materials and supplemental Fig. S3). When normalized to PC (to define the relative lipid composition of the HDL subclasses), baseline profiles of the molar ratios of lipid classes showed small dense HDL3c to be in discontinuity with all other subclasses as exemplified by peak values for both core (CE, TAG) and surface lipids (polar LPC, LPC(O), LPE, LPI, and amphipathic DAG and dhCer), which were up to threefold superior to those in HDL2b,2a, 3a, and 3b (*P* values up to *P* < 0.001) (Figs. 1B–D, H, I, M, O, Q and 2B–D, H, I, M, O, Q). Likewise, the lipidomic profile of HDL2b was distinguished by a discontinuation with all other subclasses due to peak particle abundance of specific surface components (COH, SM, and amphipathic glycosphingolipids) (Figs. 1A, E, R–U and 2A, E, R–U). Thus, molar ratios of SM/PC (≈0.2:1) were 1.4-fold elevated in HDL2b relative to HDL3, b, and c (Fig. 1E).

Six distinct lipid profiles expressed on a per particle basis (normalized to PC) were identified at baseline across HDL subclasses: (i) a predominantly monotonic profile from HDL2b to HDL3b, with a point of inflection to a peak value in HDL3c, as noted above, (ii) a peak ratio in HDL2b with lower values across the denser HDL subclasses as noted above, (iii) an overall concave profile with maxima in HDL2b and HDL3c, as observed for Cer, THC, and LPC(O) (Figs. 1 and 2P, T, I), (iv) a profile showing a progressive increase in lipid/PC ratio from large to small HDL, as exemplified by phosphatidylethanolamine and PE(P) (Figs. 1 and 2J, K), (v) a predominantly monotonic profile as seen for PE(O) (Figs. 1 and 2L), and (vi) a moderately convex profile as exemplified by PC(O), PC(P), and PI, with the lowest molar ratios at each extremity of the HDL density and size distribution (Figs. 1 and 2G, F, N, respectively). Such lipidomic profiles attest to distinct metabolic and structural constraints, which underlie the particle abundance of each lipid class in individual HDL subclasses.

The profile for the core CE/TAG ratio was monotonic within a range of 6–7:1 mol/mol (Figs. 1 and 2V); the lower values estimated by chemical analysis above reflect the inclusion of partial glycerides (notably DAG) when TG is measured enzymatically.

Lipid profiles normalized to apoAI across HDL subclasses at baseline were consistent with those normalized to PC, with the exception that molar ratios for CE, TAG, DAG, LPC, LPC(O), and Cer/apoAI were amplified specifically in HDL2b as a result of the highest lipid abundance/apoAI ratio among HDL particle subclasses (supplemental Fig. S4B–D, H, I, M–Q) (51, 52).

Overall, these findings highlight differences between the lipidome of large HDL2b relative to that in smaller HDL3a, 3b, and 3c in HTG subjects at baseline. Additionally, marked difference in particle abundance of specific core and surface lipid classes/PC distinguished small HDL3c. These findings substantiate clear differentiation of the transport and metabolism of specific lipid classes among HDL subfractions in HTG. Such marked differences involve both the surface lipid mosaic monolayer and core lipid composition.

Poststatin lipidomic profiles for TAG, DAG, LPC, LPC(O), PE, and PI/PC showed a trend to decrease across HDL subclasses, whereas those of SM, PC(P), PC(O), PE(P) PE(O), and GM3/PC trended to increase (Fig. 1). Profiles for COH, CE, Cer, dhCer, MHC, DHC, and THC/PC were essentially unchanged.

**Fig. 3:**
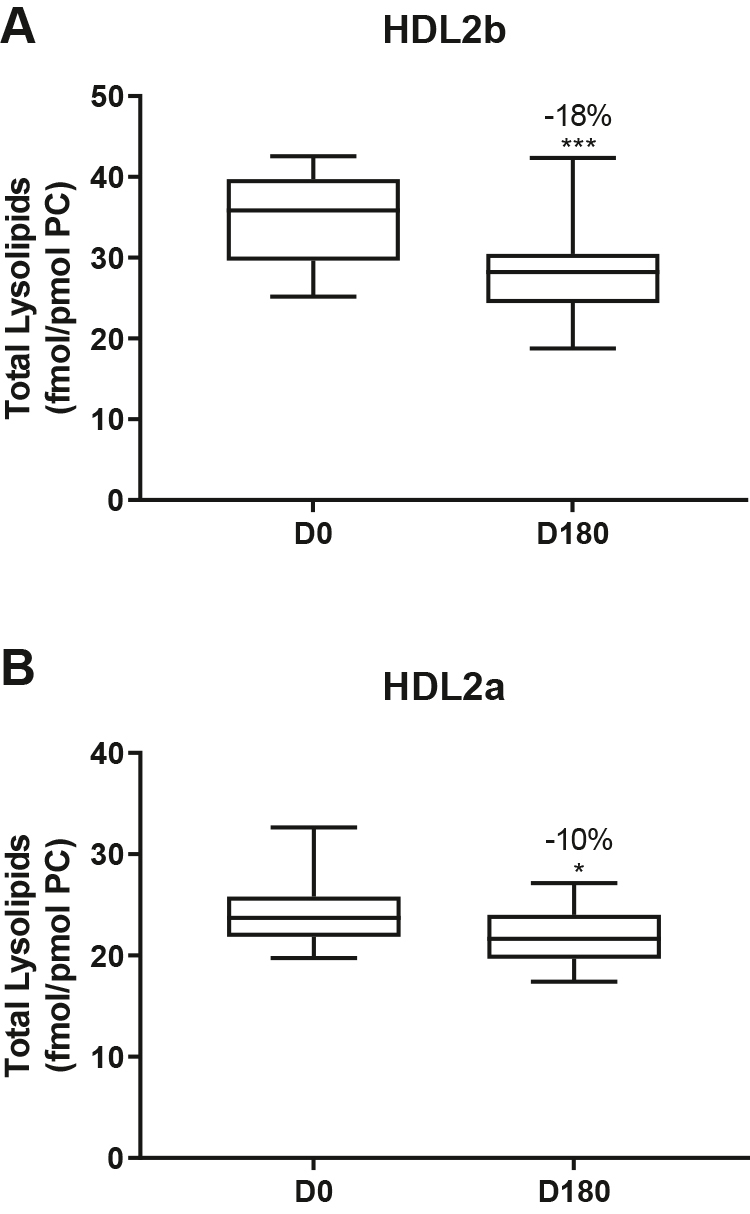
The effect of pitavastatin calcium treatment (4 mg/day; D0) for 180 days on baseline plasma concentrations of total lysolipids in the HDL2b (calculated as the sum of LPC, LPC(O), LPI, and LPE) (panel A) and in the HDL2a (panel B) subclsses from hypertriglyceridemic subjects. Data are expressed as means ± SEM (n = 12) in pmol of lysolipids/pmol PC. Percent change (%) was calculated relative to baseline values (D0). ∗∗∗*P* < 0.001; ∗∗0.001 < *P* < 0.01, and ∗0.01 < *P* < 0.05 versus D0. Density ranges: HDL2b = 1.063–1.091 g/ml; HDL2a = 1.091–1.110 g/ml. LPC, lysophosphatidylcholine; LPC(O), lysoalkylphosphatidylcholine; LPI, lysophosphatidylinositol; LPE, lysophosphatidylethanolamine.

Statin treatment mediated substantial reduction in bioactive LPC/PC molar ratio in small HDL3c (−57%) (Fig. 1H and supplemental Fig. S4H); particle abundance of LPC(O), LPE, and LPI/PC equally diminished markedly in this subclass (−45% to −52%) (Fig. 1I, M, O). Lesser degrees of reduction were observed in LPC/PC and LPC/apoAI ratios (−12% to −19%) in the largest particles, HDL2b and 2a; similar reductions (−10% to −18%) occurred in the sum of the lysophospholipids, LPC, LPC(O), LPI, and LPE/PC in both these subclasses (Fig. 3A, B). Plasma LpPLA2 activity was positively correlated with both total LPC/PC and total lysolipids/PC in HDL2b (both *P* < 0.001) poststatin, suggesting that it may contribute to the HDL lysolipid pool. Remarkably, particle abundance of Cers and related sphingolipids (dhCer, MHC, DHC, THC, GM3/PC) was refractory to statin treatment, except for minor increments of GM3 and THC in HDL2a, and of Cer in HDL3a (+9 to +18%) (Fig. 1P–U). In contrast, SM, PC, Cer, dhCer, MHC, DHC, GM3/apoAI decreased in HDL2b poststatin (up to −25%; *P* values < 0.01) (supplemental Fig. S4E, F, Q–U).

Poststatin, significant depletion in core TAG/PC and DAG/PC occurred across subclasses (−21% to −40%) (except TAG/PC in HDL3b) (Fig. 3C, D). Consequently, major particle core enrichment in CE (as CE/TAG; up to +48%; Fig. 1V) was confirmed poststatin (except HDL2b). Consistent reduction (12%–15%) in anionic PI particle abundance occurred in all subclasses (except HDL2a) (Fig. 1N). Finally, statin treatment induced a trend to enrichment (up to 30%; *P* for trend *P* < 0.05) in both plasmalogens, PC(P) and PE(P)/PC in all HDL2 and HDL3 subclasses; however, such increments were not significant in individual subclasses (Fig. 1F, K).

In summary, statin-mediated remodeling of all bioactive surface lysophospholipids was most pronounced in small HDL3c; in contrast, baseline particle abundance of Cer, dhCer, and all glycosphingolipids was maintained across subclasses following statin treatment. With minor exception, molar ratios of TAG/PC and DAG/PC were diminished poststatin, with a strong trend to normalization of core CE/TAG ratios.

### HDL subclasses: molecular lipid species at baseline in HTG subjects and effect of pitavastatin treatment

Sixty-one molecular species quantitatively predominated (each >5% of total in individual lipid classes) among the 23 lipid classes across HDL subclasses. Poststatin, the distributions of lipid species were altered as follows: (i) in HDL2b, CE (CE(16:0), CE(18:1), and CE(18:2) (up to −18%; *P* < 0.003) (data not shown), (ii) reduction in four predominant species of TAG (up to −42%; *P* values <0.02) in all subclasses except for HDL3b (supplemental Table S3), (iii) reduction in six prominent species of DAG ((16:0/18:1); (16:0/18:2), (16:1/18:1), (18:0/18:1), (18:1/18:1), and (18:1/18:2; up to −41%; *P* values <0.03) in all subclasses with minor exception (data not shown), (iv) PC, with increment in PC(38:4) (+16 to +23%; *P* < 0.006 or less) across all subclasses, (data not shown), (v) SM, in which the predominant SM, SM(34:1) (≈30% of total), increased poststatin in HDL2a, 3a, and 3b (up to 19%; *P* < 0.04 or less) (data not shown), (vi) Cer-related glycosphingolipids, in which significant increment in the C16:0 saturated species was observed in MHC, DHC, and THC (range +17 to +29%; *P* < 0.05 or less), with enrichment in Cer(16:0) and Cer(22:0) in HDL3a (+16 and +12%, respectively; *P* < 0.05) (data not shown), and (vii) two saturated LPC species, LPC(16:0) and LPC(18:0), (>60% of the LPC lipidome across all subclasses), in which marked reduction (up to −55%; *P* < 0.05 or less) occurred in all subclasses, except for HDL3c (LPC(18:0), NS) (data not shown). Abundance of LPC(16:0) and LPC(18:0) (>80% of total) in HDL2b was positively correlated with LpPLA2 activity poststatin (both *P* < 0.001). HDL2a content of total lysophospholipids/PC, together with that of both the LPC(16:0) and LPC(18:0) species, was correlated with LCAT activity (all 0.01 < *P* < 0.05 and 0.001 < *P* < 0.01, respectively).

Overall, statin treatment mediated marked remodeling of molecular species of core CE and TAG, and of bioactive surface lipids (LPC, PC, SM, Cers and related sphingolipids, and DAG) across HDL subclasses.

### Total HDL: proteomic analyses in HTG subjects at baseline and effect of pitavastatin treatment

**Fig. 4:**
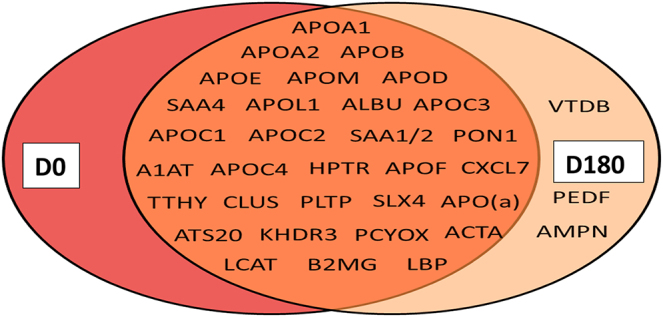
Venn diagram showing proteins detected by MS/MS across all HDL subclasses at baseline (D0) and postpitavastatin treatment (D180) in hypertriglyceridemic subjects; detection was based on the identification of at least two identified peptides in a Mascot analysis using Scaffold (see Materials and Methods). Forty unique proteins were detected across all HDL subclasses. Human keratins K2C1, K1C10, K22E, K1C9, and K1C14 were also detected but not included, as they likely reflect contaminants from sample handling. Proteins unique to D180 do not necessarily reflect statin-induced proteins because these tended to be close to the lower limit of detection and were not found to be statistically different from zero in some cases. Albumin (ALBU), serum amyloid 4 (SAA4); serum amyloid 1/2 (SAA1/2); α-1-anti-trypsin (A1AT); haptoglobin-related protein (HPTR); chemokine ligand-7 (CXCL7); transthyretin (TTHY); clusterin or apoJ (CLU); structure-specific endonuclease 4 (SLX4); ADAM metallopeptidase with thrombospondin type 1 motif 20 (ATS20); KH domain-containing, RNA-binding, signal transduction-associated protein 3 (KHDR3); prenylcysteine oxidase 1 (PCYOX); actin assembly–inducing protein (ACTA); β-2-microglobulin (B2MG); vitamin D–binding protein (VTDB); pigment epithelium-derived factor (PEDF); and aminopeptidase N (AMPN).

Initial proteomic analyses were designed to detect statin-driven changes in the total protein content of HDL, rather than reflecting whole body changes in HDL protein levels (i.e., equal total protein masses were analyzed by LC-MS, irrespective of the quantities present in each subject). Consistent with data in the literature for individual HDL isolates, 40 unique HDL proteins were identified at baseline (16); five were human keratins which are common contaminants from sample handling, resulting in 35 that were likely specific to the HDL samples (Fig. 4). Of these, 32 proteins were detected in our total HDL fractions both prestatin and poststatin treatment. No proteins were unique to baseline T0 samples, while three were unique to poststatin samples (vitamin D–binding protein (VTDB), pigment epithelium-derived factor (PEDF), and aminopeptidase M (AMPN). All detected proteins had previous entries in the HDL Proteome Watch Database (16).

**Fig. 5:**
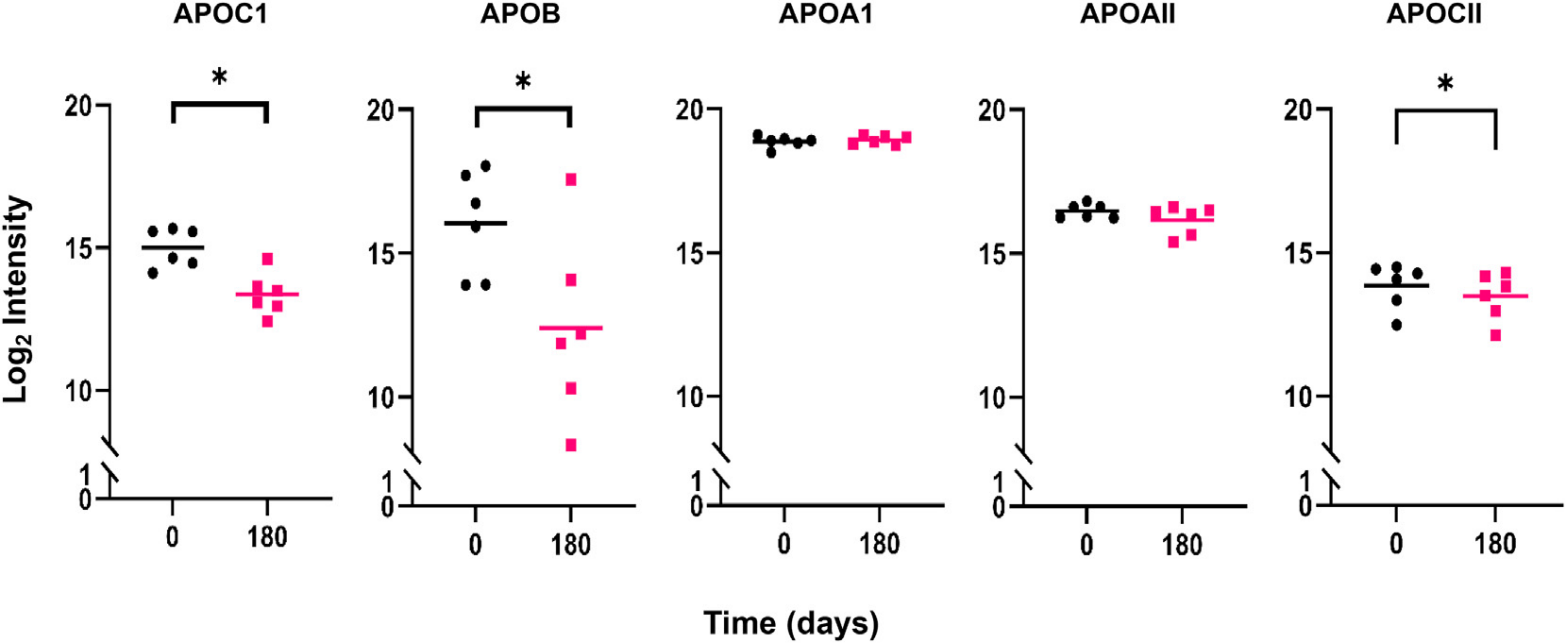
The effect of pitavastatin calcium treatment (4 mg/day) on apoCI, apoB, apoAI, apoAII, and apoCII abundance in the proteome of the total HDL fraction (d 1.063–1.21 g/ml) hypertriglyceridemic subjects. HDL proteins were quantified using MaxQuant and LFQAnalyst as described in Methods (Supplementary Material). Proteins that underwent significant change poststatin, that is, apoCI and apoB, are shown alongside the unchanged scaffold proteins, apoAI and apoAII; the distribution of the LFQ intensity for each of the four proteins in the total HDL fraction is presented for each individual at baseline (D0) and poststatin (D180), with the horizontal bar representing the mean value. ∗*P* < 0.05 using a paired *t* test with correction for multiple comparisons. ApoCII was manually quantified using LFQ intensities from MaxQuant after selecting apoCII-specific peptides that were part of the apoCIV-apoCII identification cluster. Three of four peptides quantified by MaxQuant belonged to apoCII with the other mapping to apoCIV. The data represent the total apoCII protein intensity for each individual analyzed, with the horizontal bar showing the mean value. Statistical analysis of apoCII data was performed using a paired *t* test without correction for multiple comparisons (∗*P* < 0.05). LFQ, label-free quantitation.

To gain insight into possible statin-induced changes in protein abundance in total HDL, a LFQ analysis was performed (see Supplementary material). A reliable MaxQuant quantitation of 13 of the 35 detected proteins was obtained. The major HDL scaffold proteins, apoAI and apoAII, did not differ prestatin and poststatin treatment with respect to overall HDL protein content, indicating that total protein levels in samples for MS/MS analysis were well matched. Most detected proteins showed no significant differences with respect to statin treatment. However, apoCI and apoB were both reduced (≈66% and ≈ 68%, respectively; each *P* < 0.05) poststatin (Fig. 5). By manual analysis, apoCII was also diminished (≈30%; *P* < 0.05) poststatin consistent with data in whole plasma (Table 1 and Fig. 5 and legend). For proteins that could not be rigorously quantitated by the MaxQuant algorithm, an MS1 full-scan filtering analysis was performed using Skyline. This analysis confirmed reductions in apoCI, apoCII, and apoB identified by MaxQuant but also suggested statin-driven reductions in apoM (reduction ≈ 22%) and apoCIV (reduction ≈ 61%) (data not shown).

### HDL subclasses: proteomic analyses in HTG subjects at baseline and effect of pitavastatin treatment

**Fig. 6:**
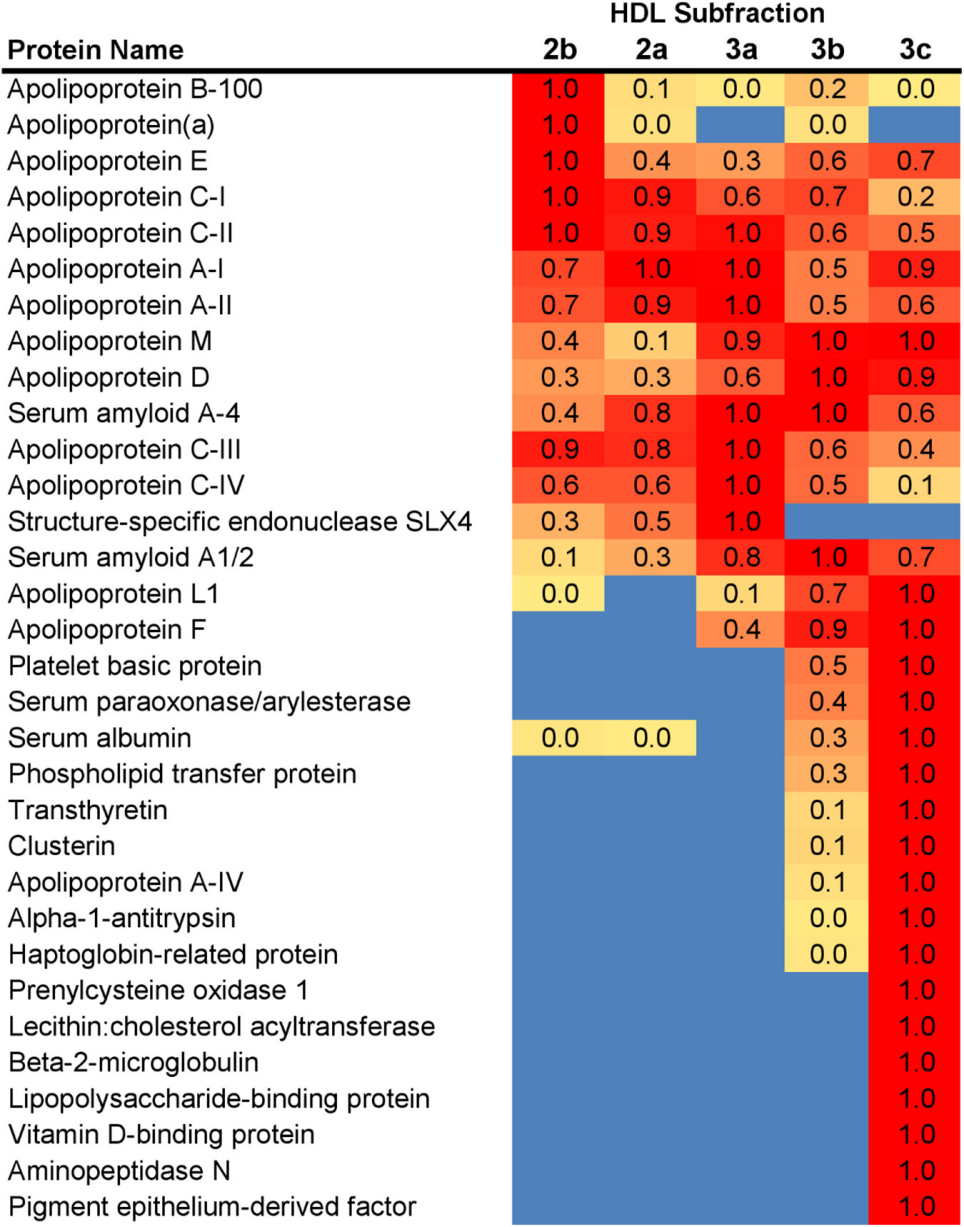
Heat map of protein distribution patterns across HDL density subclasses in hypertriglyceridemic subjects. HDL subclasses are indicated at the top. The proteins identified by Scaffold peptide analysis are listed at left. The spectral counts (i.e., the total number of spectra identified for all peptides within a given protein) were determined for each protein in each fraction; data for D0 and D180 time points were combined given the minor differences in total proteome of HDL between them. The fraction that contained the highest peptide count for a given protein was normalized to 1 and all other values for that protein were scaled accordingly. The highest relative values are colored red and graduate to yellow for the lowest values (blue indicates that no peptides were found in any sample). Proteins were ordered in terms of preference from least dense to most dense based on the peak fraction.

Prestatin and poststatin, HDL3c displayed marked protein diversity, with 20 proteins in greatest abundance among subclasses (Fig. 6); nine proteins (PEDF, AMPN, VTDB, lipopolysaccharide-binding protein, β-2-microglobulin, LCAT, prenylcysteine oxidase 1, haptoglobin-related protein, α-1-antitrypsin (A1AT) were unique to HDL3c, while a further three (apoJ, apoAIV, and transthyretin) were most abundant in HDL3c with traces in HDL3b. ApoB content in light HDL2b, in which apo(a) was uniquely detected, decreased poststatin (supplemental Fig S5). ApoB detection may derive from minor amounts of Lp(a) given its immunodetection in HDL2b, and/or from traces of dense LDL, and/or from association with proteolytic fragments (34, 53). Poststatin, no consistent changes in other quantifiable proteins, including apoAI, apoAII, apoCI, apoCIII, apoE, apoD, apoM, apoL1, SERPINA1, and SAA2-SAA4, were detected in individual HDL subclasses (supplemental Fig S5).

## Discussion

The present studies represent the first comprehensive quantitative and qualitative characterization of the lipidomic profiles of five, physicochemically defined HDL subclasses in nondiabetic, hypertriglyceridemic, hypercholesterolemic obese males at baseline and following treatment for 180 days with pitavastatin calcium (4 mg/day). The overall treatment effect on the baseline lipidome was evaluated by comparison of total HDL prestatin and poststatin, and in turn, with the corresponding lipidome in control subjects. Bioactive lipids relevant to the biological activities of HDL were of paramount interest. Lipidomic profiles were complemented by proteomic arrays of both total HDL and its constituent subclasses prestatin and poststatin, allowing an integrated vision of the impact of statin treatment on the proteolipidome of HDL and its subclasses in HTG.

Several salient findings emerged at baseline: (i) HTG was associated with profound and specific depletion of some 21 lipid classes in total HDL (−40% or more), relative to controls (normalized to apoAI), (ii) six distinct lipidomic profiles were identified across subclasses/PC, (iii) the lipidome of small, dense HDL3c was distinguished by peak particle abundance of specific surface and core lipids (lysophospholipids, amphipathic DAG and dhCer, and core CE and TAG, normalized to mol PC), whereas peak particle abundance of COH, SM, glycosphingolipids (MHC, DHC, THC, and anionic GM3) characterized large, light HDL2b. Thus, HDL2b and HDL3c particles were clearly differentiated from each other and from medium-sized subclasses (HDL2a, HDL3a, and 3b) by marked discontinuities in lipid profile/PC, (iv) both SM/PC and COH/PC molar ratios (≈0.2/1 and ≈0.45:1, respectively) peaked in HDL2b (Figs 1E and 2E) as compared to those in HDL3c (SM/PC and COH/PC, ≈0.14:1 and ≈0.21:1), (v) core CE/TAG ratios varied over a narrow range (6–7:1 mol/mol) from HDL2b to HDL3c, (vi) all metrics of HDL plasma levels were subnormal, including concentrations of the scaffold proteins, apoAI and AII, and (vii) dense HDL3c was distinct in its marked protein diversity, and was further distinguished by nine unique proteins with distinct biological activities, PEDF, AMPN, VTDB, lipopolysaccharide-binding protein, β2MG, LCAT, prenylcysteine oxidase 1, haptoglobin-related protein, and A1AT.

Highlights of lipidomic and proteomic changes mediated by pitavastatin calcium therapy featured: (i) a marked overall trend to normalization of the lipid-poor (total) HDL lipidome on a per particle basis (normalized to apoAI) in HTG subjects, with the exception of reduction in elevated baseline abundance of both core TAG and amphipathic DAG (−19% and −32%, respectively; NS) (Table 2), (ii) pronounced particle remodeling of bioactive surface lysophospholipids (LPC, LPC(O), LPE, and LPI) in small HDL3c (range from −45% to −57%), in contrast to minor reduction (HDL2b and 2a; range from −12% to −21%) or no change in these lipids in other subclasses (values normalised to PC), (iii) maintenance of baseline lipidomic profiles of Cer, dhCer, and related glycosphingolipids and GM3/PC across particle subclasses with minor exception, (iv) pronounced remodeling of molecular species of core CE and TAG, and of bioactive surface lipids (LPC, PC, SM, and DAG) across HDL subclasses, (v) marked depletion in TAG/PC, resulting in relative core enrichment in CE (NS for reduction in TAG/PC in HDL3b), and equally in DAG/PC ratios across subclasses, (vi) remodeling of the (total) HDL proteome with diminished abundance of apolipoproteins CI, CII, CIV, and M, (vii) reduction in apoB abundance, reflecting diminution in either Lp(a)-apoB, dense LDL-apoB, or apoB peptides or a combination thereof, and (viii) maintenance of elevated proteomic diversity in HDL3c.

### Lipidomic profiles of HDL particle subclasses in HTG: focus on bioactive lipids

The peak of lysophospholipid abundance specific to small, dense HDL3c particles in HTG subjects at baseline indicates that a uniform ratio corresponding to an equilibrium state across subclasses was not established, thereby favoring validation of the isolation procedure. Further, this finding is of immediate relevance to HDL metabolism and to the pathophysiology of atherosclerosis. A primary source of LPC in HDL3c is potentially LCAT activity not only in light of the presence of this protein in the HDL3c proteome but also in view of the peak particle abundance of the product of the LCAT reaction, CE, in HDL3c, both prestatin and poststatin. Indeed, Pearson correlation data suggest that both LCAT and LpPLA2 contribute to regulation of LPC content in HDL2 and HDL3 particles. Equally, LPC in HDL may originate from LPC-enriched VLDL, with lipolytic liberation of LPC-rich surface fragments which then sequester to HDL via PLTP-mediated transfer (8). Further, HL and EL activities, known to be elevated in HTG, may contribute to HDL LPC formation (6, 7). Moreover, LPC may arise from the action of HL on apoAII-containing HDL2, with conversion to HDL3 in the postprandial period (54).

The lower ratios of SM/PC and COH/PC in HDL3c relative to those in large HDL2b, (molar ratios: ≤ 0.14:1 for SM/PC and ≈0.2:1 for COH/PC in HDL3c vs. 0.2:1 and 0.45:1, respectively in HDL2b; *P* < 0.001 vs. HDL2b), both prestatin and poststatin, are consistent with the postulate that surface lipid monolayer rigidity is greater in HDL2b than in HDL3c (55); further, tighter PL packing and slower surface dynamics are characteristic of high SM/PC ratios, a feature amplified by elevated COH content as in HDL2b (55). In this light, surface located, hydrophilic LPC, LPE, and LPI (molar ratios of 0.1:1, 0.002:1, and 0.01:1/PC, respectively) in HDL3c would be anticipated to preferentially engage in exchange and/or transfer with cell membranes, as well as with other lipoprotein particles (e.g., VLDL/IDL) and albumin-rich very high–density lipoproteins; indeed, VLDL/IDL exhibit a low SM/PC ratio with high surface fluidity (35). Particle lipid abundance/PC does not however provide insight into the flux of lysophospholipids through these particles.

LPCs exert multiple biological activities, including activation of oxidative stress and proinflammatory responses (56, 57). Transendothelial transport of HDL with entry into the subendothelial space facilitates local diffusion of HDL-associated LPCs and other lysophospholipids into the aqueous phase, potentiating sequestration in cell membranes of arterial wall cells, or those of the plaque, or both (56, 58). Both LPC and LPI activate a spectrum of intracellular, proinflammatory signaling pathways via binding and activation of G protein–coupled receptors (56, 57, 59). Indeed, LPI is a functional ligand for the GPR55 receptor, which activates multiple signal transduction pathways and is implicated in regulation of endothelial function, angiogenesis, cell proliferation, migration, and survival (59). Data in human plaques suggest that LPCs are implicated in plaque inflammation and vulnerability (60). Further, LPC is a substrate for autotaxin, a secretion product of vascular interstitial cells; autotaxin exerts lysophospholipase D activity, thereby mediating formation of lysophosphatidic acid (LPA). LPA, a potent signaling ligand, may in turn activate the LPA receptor on interstitial cells, leading to a nuclear factor KB-mediated inflammatory cascade (61). The physiological functions of LPE have not yet been fully elucidated.

The peak particle abundance of core TAG in HDL3c at baseline (≈0.15 mol/mol PC vs. ≈0.007–0.009 mol/mol PC in other subclasses; *P* < 0.001) and equally of amphipathic DAG (≈0.045 mol/mol PC vs. ≈ 0.025–0.028 mol/mol PC in other subclasses; *P* < 0.001) is consistent with the preferential transfer of TRL-derived TAG and DAG to this subclass, and with the HL-mediated lipolytic formation of DAG from TAG within these particles (6, 8). Elevated rates of CETP-mediated heteroexchange and transfer of TAG from TRL to HDL are characteristic of HTG states (62). Lipolytically-derived surface fragments of TRL may equally contribute to both TAG, and potentially DAG, enrichment of HDL (8). Further, TAG-enriched HDL in HTG is a ready substrate for HL, a further source of DAG (6). As a consequence of their amphipathic nature, DAGs partition between the surface and lipid core of HDL and may diffuse out into the aqueous phase (63). Plasma membrane insertion would then facilitate DAG action as a second messenger, in which case DAG species can activate protein kinase C isoforms with downstream protein phosphorylation (63).

Interestingly, abundance of DAG in HDL subclasses (≈0.02-0.03:1 mol/mol PC) is similar to that in LDL subclasses in this same cohort, as is the case for TAG (LDL subclasses, ≈ 0.1:1 mol/mol PC; HDL subclasses, 0.07–0.09 mol/mol PC, but excluding HDL3c) (35).

### Statin-mediated remodeling of lipidomic profiles of HDL subclasses in HTG

Pitavastatin treatment exerted targeted remodeling of the HDL subclass lipidome in HTG, modifications primarily concerning lysophospholipids, core TAG, amphiphilic DAG, PI and plasmalogens on a per particle basis. Major statin-mediated reductions in abundance of all lysophospholipids, i.e. LPC, LPC(O), LPE and LPI/PC (range −45% to −57%) distinguished HDL3c; lesser reductions (−12% to −19%) were observed in LPC in large light HDL2b and 2a. Such decrements may in part reflect diminished LpPLA2 mass and activity post-statin (34). The direct inhibition of EL activity by pitavastatin (>50%) is equally anticipated to contribute to a major degree to overall reduction of lysophospholipid content across HDL subclasses (64). The possibility that marked statin-mediated depletion in lysophospholipid abundance/PC in HDL3c may attenuate its potential proinflammatory action is indeterminate.

Interestingly, although HDL subclasses were richer in PI than those of LDL, (LDL, ≈0.04 mol/mol PC; HDL, ≈0.075–0.090 mol/mol PC) (35), post-statin diminution of particle abundance/PC of bioactive anionic PI was similar (≈−15% in most subclasses) across both, suggesting that similar underlying mechanisms may be operative. PIs exert a wide spectrum of biological activities, including regulation of lipid dynamics in cell membranes (65). It is therefore relevant that both in vivo and in vitro data indicate that HDL-PI acts on cell surface ATP binding cassette transporters and the intracellular inositol signaling cascade, presenting as a major regulator of both cellular and intravascular cholesterol homeostasis (66).

Overall trends across HDL subclasses to statin-mediated normalisation of particle abundance of surface-located SM, PC(P), PE(P), PC(O) and PE(O) are consistent with findings for their abundance/apoAI in the total HDL fraction. The major plasmalogens, PE(P) and PC(P), are principally transported in HDL (≈60% of total circulating plasmalogens), and protect labile unsaturated lipids, such as unsaturated CE species and PLs containing polyunsaturated fatty acids, by scavenging reactive oxygen species. Consistent with earlier findings of reduced abundance of plasmalogens in total HDL2 and HDL3 at baseline in the CAPITAIN cohort, significant trends to enhanced content across all five subclasses were observed post-statin (p for trend <0.05) (36). Whether this effect results from statin-mediated upregulation of plasmalogen synthesis in hepatic peroxisomes via PPAR activation cannot be excluded; indeed, PPAR activation has been suggested to represent a component of the pleiotropic action of statins (67). Equally however, statin-induced attenuation of systemic oxidative stress, typical of HTG states, may spare HDL plasmalogens (68, 69).

Following marked statin-mediated reduction in TG levels, substantial depletion of core TAG/surface PC ratio was found in HDL subclasses (except for HDL3b) (Fig. 3C), an effect likely representing a metabolic consequence of enhanced statin-driven TRL clearance, with reduction in TAG substrate for CETP-mediated transfer to HDL (3, 62). In turn, diminished TRL-TAG substrate availability for DAG formation by lipolysis may underlie significantly lower DAG particle abundance across all HDL subclasses (Fig. 3D).

As minor components of the HDL lipidome, Cer, its immediate precursor, dhCer, and the glycosphingolipids, MHC, DHC, THC, and GM3 represented 0.004 mol/mol PC or less across HDL subclasses, abundances which were essentially unchanged by statin treatment and suggestive of preferential conservation. Total HDL subclass Cer concentrations were ≈0.005 mol/mol PC, some 6-fold less than that transported in LDL subclasses in these HTG subjects (35). Such elevated levels of lipotoxic Cer in LDL particles have been associated with multiple mechanisms underlying major adverse atherosclerotic cardiovascular events and in addition, with impaired glucose homeostasis, insulin resistance, inflammatory processes, and cellular stress responses (70). Glucosylated Cers (e.g., GM3) also contribute to cardiometabolic disease, in part by inhibiting insulin signaling (70). Functionally, these lipids appear to be critical to membrane lipid microdomain structure and potentially to that of the HDL surface lipid monolayer. It is however indeterminate as to whether HDL-associated Cers and related sphingolipids may be taken up by blood or tissue cells and whether deleterious cellular effects may ensue.

### Statin-mediated proteomic remodeling of HDL and its subclasses

Proteomic data in HTG HDL subclasses resemble previous findings in control subjects, which indicated that exchangeable proteins are in disequilibrium across subclasses, with the smallest, densest particles exhibiting the greatest proteomic diversity (17). Large HDL2b and 2a shared an elevated abundance of a cluster of three metabolically significant apolipoproteins, apoCI, apoCII, and apoCIII (Fig. 6). Furthermore, apoE, a key ligand of the LDL receptor, exhibited elevated particle abundance in both HDL2b and HDL3c (Fig. 6). Predictably, metabolic studies revealed apoE to be a key determinant of the plasma residence of HDL particles, influencing both delivery of cholesterol cargo to the liver and particle expansion (71). By contrast, the presence of apoCIII on an apoE-containing HDL subspecies counteracts the effect of apoE, attenuating particle expansion and inhibiting clearance (71, 72). Small HDL3 particles were also enriched in proteins like apoM, the specific transporter for S1P (26).

Interestingly, and compared to lipid classes, the HDL subclass proteome was not strikingly altered by pitavastatin treatment. Of the 32 proteins detected prestatin, a small number of proteins underwent statin-mediated remodeling, with significant loss of apolipoproteins CI, CII, M, and CIV (−68%, 30%, −22%, and −61%, respectively). Poststatin reduction in HDL-associated apoCII contributed to that in plasma (−61%; *P* < 0.001) and occurred concomitantly with fall in apoCII-containing remnant levels (as RLP-C, −55%) (4) (Table 1). Further, statin-mediated reduction in apoM may explain the corresponding decrease in S1P levels discussed above. These data are consistent with those in moderately hypertriglyceridemic/low HDL-C subjects (plasma TG, 170 mg/dl) on atorvastatin monotherapy in the Cardiac Plaque Composition study in which significant reductions in apoCI, apoCII, apoCIII, and apoCIV (all > −15%), apoE (−21%), apoM (−13%), and apoL1 (−4%) were observed (73). As statins accelerate the catabolism of TRL, in part by enhancing hepatic removal in vivo, the plasma pool of TRL-associated C apolipoproteins is predicted to diminish concomitantly (5). Whether statins exert more direct effects on lipolytic enzymes and C apolipoproteins implicated in TRL metabolism remains conjectural; neither atorvastatin nor rosuvastatin exert discernible effects on lipoprotein lipase activity, although HL activity diminished (up to ≈20%) over an 8-week treatment period with these agents (29, 30). Furthermore, these statins were without significant effect on both apoCI and apoCIII levels (30).

In earlier studies, several proteins implicated in lipid transport and metabolism, complement regulation and the acute phase were reported to either increase or decrease in the HDL proteome after short-term treatment (28 days) with rosuvastatin in healthy subjects acting as their own controls (74). Remarkably, more than a 5-fold increase was observed in A1AT in large (HDL2-like) HDL, resulting in enhanced antiprotease activity. Several factors may underlie such contrasting results: (i) our analyses were normalized to protein content, thereby attenuating signals indicative of relative changes in abundance of component proteins, (ii) the baseline lipid phenotype of the study cohort, (iii) the selected statin together with dose, the period of statin treatment and the degree of modulation of lipoprotein levels, (iv) HDL isolation and fractionation methodologies, and (v) mass spectrometric analytical methodology and related software.

### Limitations

Limitations include the restricted cohort size (n = 12) and recruitment of male subjects. Extensive phenotyping both prestatin and poststatin was however performed on a background of rigorous inclusion and exclusion criteria (see Methods and Supplement). Volunteers displaying nondiabetic HTG acted as their own controls, in part limiting confounding effects arising from variability in statin response linked to genotypic background (42) and from potential differences in baseline phenotype in a placebo group. Moreover, key assumptions relating to the additivity of drug-placebo effects in randomized clinical trials have been questioned (75). Regression to the mean in human studies may however lead to overestimation of treatment effects in the absence of a placebo control group. The high precision and reproducibility of the lipidomic analyses performed by LC-MS provided sufficient power to clearly identify lipid class effects, even with correction for multiple comparisons (33, 35, 76). However, a statin comparator was lacking in our clinical protocol; comparable proteolipidomic data to those presented here for pitavastatin calcium are not available for another member of this drug class. These original findings now require validation in a large prospective study over a longer period in an independent cohort of hypertriglyceridemic, low HDL-C subjects to include women, preferably with both a statin comparator and a placebo group. Focus on identification of proteome-defined HDL subspecies is essential, as the proteomic composition of each subclass represents the summed averages of the proteomes of constituent particle subspecies.

## Conclusions

In sum, marked discontinuities in both the lipidome and proteome of large light HDL2b and small dense HDL3c characterize the HDL subclass profile in nondiabetic HTG subjects on a per particle basis, distinguishing each of them from medium-sized HDL2b, 3a, and 3b. These findings are consistent with intestinal and/or hepatic secretion of stable HDL size subsets displaying discrete compositional features, and equally with particle subset-specific intravascular metabolism 73). The extensive remodeling of the HDL subclass lipidome in nondiabetic HTG, likely a consequence of defective metabolism of TG-rich lipoproteins, involved enrichment in some lipids/PC, loss of others, while yet others showed no change in particle abundance. The HDL subclass proteome was altered, but to a lesser degree than the lipidome. We formulate the hypothesis that pitavastatin calcium mediates partial normalization of HDL particle composition, thereby mirroring restorative changes in TG-rich lipoprotein metabolism. Statin therapy primarily modulates remodeling processes implicated in key metabolic mechanisms of the TRL-HDL axis, and notably CETP-mediated neutral lipid heterotransfer and exchange between TRL and HDL (29, 30, 31, 34, 62), lipolytically-driven, PLTP-mediated, transfer of surface fragments from TRL to HDL (8), LCAT-driven cholesterol esterification in HDL (77), endothelial and/or HL-driven hydrolysis of HDL PC and/or TAG (64, 78), possibly PLTP-driven HDL particle fusion, and potentially cellular processes involved in lipid synthesis, turnover or degradation, such as may be the case for plasmalogens (36). Clinically, our findings suggest that further increase in HDL-cholesterol concentration beyond statin monotherapy, to levels well within the range associated with lowest all-cause mortality (58–76 mg/dl in males), together with normalization of both composition and plasma residence time of HDL particles, is desirable in nondiabetic hypertriglyceridemic patients with low HDL-C at high cardiovascular risk (1, 4, 6, 28, 32, 62, 79). Finally, it remains indeterminate as to what degree the statin-mediated remodeling of the HDL proteolipidome might impact the defective biological activities of HTG HDL (3, 6, 11, 29, 80).

## Data availability

All data is available upon request to the corresponding author.

## Supplemental data

This article contains supplemental data (17, 22, 33, 34, 35, 36, 45).

## Conflict of interest

The authors declare that they have no conflicts of interest with the contents of this article.
