# Supplementary material for: Pitavastatin treatment remodels the HDL subclass lipidome and proteome in hypertriglyceridemia
Source: J Lipid Res. 2023 Dec 29;65(2):100494. doi: 10.1016/j.jlr.2023.100494 (PMC10850136; doi:10.1016/j.jlr.2023.100494)
Supplement: Supplementary material [file mmc1.docx]

**Supplementary Material**

**Pitavastatin treatment remodels the HDL subclass lipidome and proteome in hypertriglyceridemia.**

M. John Chapman, BSc (Hons), PhD, DSc ^1^ *, Alexina Orsoni, PhD ^2^, Natalie A. Mellett, BSc^3^, Anh Nguyen, BSc ^3^, Paul Robillard, BSc ^1^, Jonathan E. Shaw, MD ^3^, Philippe Giral, MD **^4^**, Patrice Thérond, PhD ^2^, Debi Swertfeger, PhD ⁵, W. Sean Davidson, PhD ⁶*, and Peter J. Meikle, PhD ^3^*

**Clinical study protocol.** Details of the clinical study protocol, the inclusion and exclusion criteria, and of dietary counseling and compliance of subjects (n=12) recruited into the CAPITAIN study (ClinicalTrials.gov: NCT01595828; Supplementary Figure S1) were detailed earlier (1-4). No serious adverse event was reported during the study protocol. The clinical protocol is summarized schematically in Figure S1 below.

**Supplementary figure S1:** Clinical protocol for the open-label CAPITAIN study in hypertriglyceridemic male subjects (n=12). In addition to hypertriglyceridemia, the plasma phenotype of recruited subjects involved elevated plasma levels of remnant lipoprotein-cholesterol, LDL-cholesterol and apoB, and subnormal levels of all plasma markers of HDL including HDL subclass concentrations, with the exception of pre-β-HDL (1-4) (Table 1; Suppl Table 2). Key inclusion and exclusion criteria, medications permitted during the period of the clinical study and which do not significantly impact lipid metabolism or lipid profile, and the time course of plasma lipid, lipoprotein and biomarker analyses over the 180 day duration of pitavastatin calcium treatment (4 mg/day) are indicated.


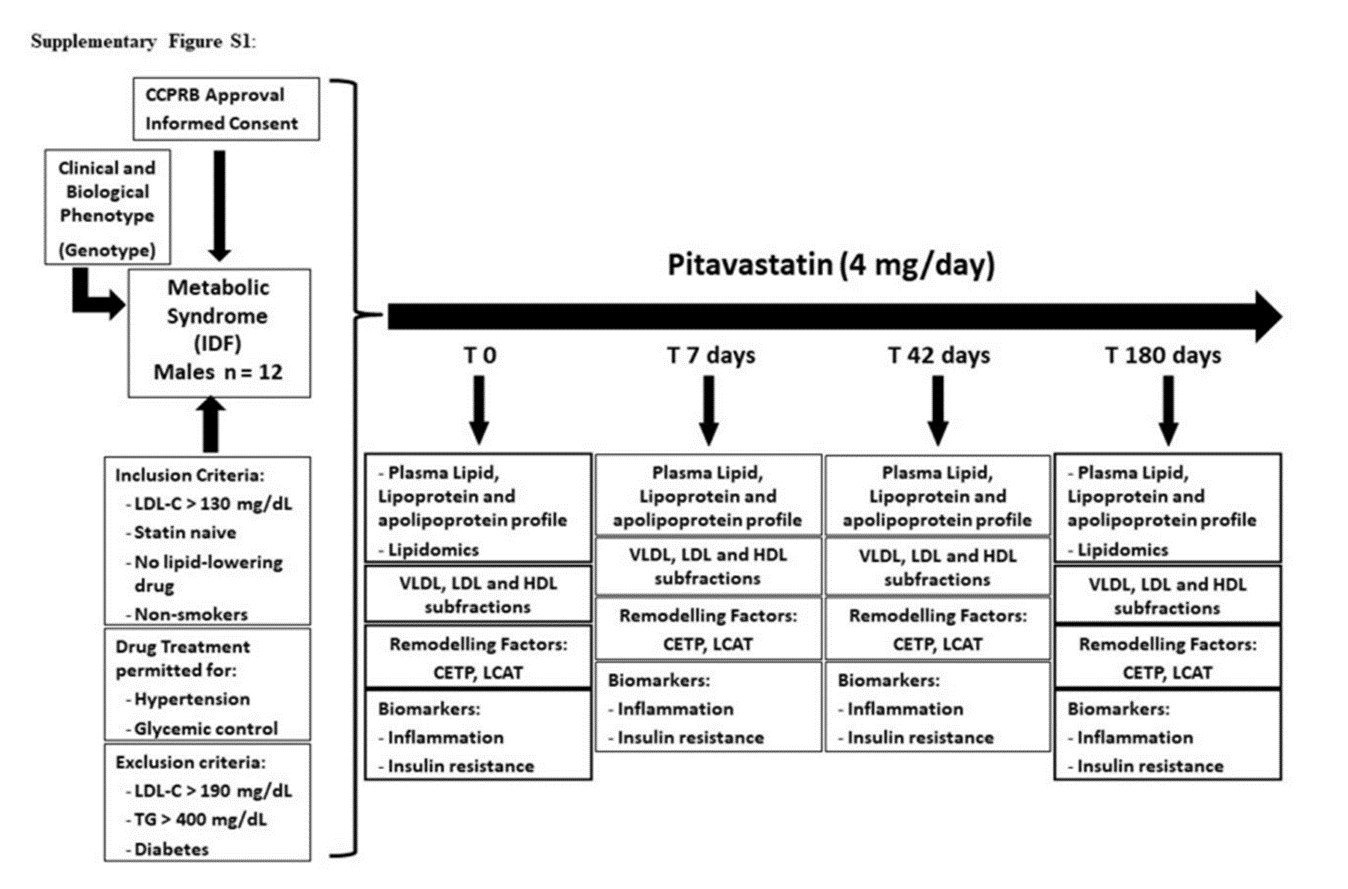


**Materials and Methods**

**Preparative methods: Density gradient isolation of HDL subclasses.**  Prior to ultracentrifugal isolation of plasma samples from subjects in the CAPITAIN cohort, blank salt gradients were ultracentrifuged, and the density profile determined to establish the specific profile; variation in absolute densities at each point along the length of the ultracentrifugal tube can vary up to the third decimal place between separate experimental series (5). Such controls ensured the accuracy and absolute values of the density intervals in which the five HDL subclasses were isolated.

Several factors may introduce variability into the isolation process and so alter both the precision and reproducibility of lipoprotein isolation, and are as follows:

-Failure to respect the rigorous conditions for gradient construction, ultracentrifugation, and gradient fractionation of HDL (2-7).

-Failure to respect rigorous conditions for preservation of the native state of plasma and for freezing and thawing of samples. For cryoprotection, sucrose (final concn 0.06%) must be present; EDTA (1mg/mL plasma) is equally required to chelate metal ions and inhibit oxidation (PMID: 27581680). Rapid freezing of each sample in thick-walled pre-sterilised tubes to -80 °C is performed in liquid nitrogen. Plasma samples can only be thawed once to +4 degrees C (in a water bath at 37 degrees C). Small aggregates of fibrin can occasionally be observed; they are not removed prior to centrifugation to avoid plasma losses and typically sediment to the bottom of the gradient tube upon isolation. Plasma samples handled according to our protocol both before and after centrifugal fractionation do not contain detectable levels of lipid hydroperoxide oxidation products.

If the conditions of plasma storage are not respected, denaturation of lipoproteins occurs with formation of aggregates, leading to artefactual data.

To address potential variability in HDL isolation, six plasma samples were randomly selected and each fractionated twice in independent ultracentrifugal runs; the variability in both quantitative and qualitative metrics (using the % weight chemical mass of each subclass as the principle criterion) was < 10% for all parameters. We equally compared lipoprotein fractions from the same plasma samples (n=3) fractionated in duplicate in the same run, with similar findings.

**Analytical methods. Lipidomic analyses by Tandem Mass spectrometry.** Lipidomic analyses of lipid extracts of HDL subclasses, and from the total HDL fraction at baseline (D0) and post-statin (D180), were performed by high performance liquid chromatography followed by electrospray ionisation-tandem mass spectrometry (LC-MS) using an Agilent 1200 liquid chromatography system combined with an Applied Biosystems API 4000 Q/TRAP mass spectrometer with a turbo-ion spray source (350°C) and Analyst 1.5 data system. The methodology, instrumentation and internal standards were identical to those used in earlier studies; indeed, the conditions for tandem mass spectrometric analysis of lipoprotein lipid classes and subclasses and the parameters of the assay performance were derived from a quality control plasma pool as detailed earlier (Supplementary Table S1) (1,2,4,8). A total of 308 molecular lipid species were analysed. Technical quality control samples were included throughout the run in every 20-sample interval. The following individual lipid classes and subclasses were analysed in each lipoprotein subfraction and quantitated: cholesteryl ester (CE), free cholesterol (COH), sphingomyelin (SM), PC, alkylphosphatidylcholine (PC(O)), alkenylphosphatidylcholine (PC(P)), lysophosphatidylcholine (LPC), lysoalkylphosphatidylcholine (LPC(O)), (lysoplatelet activating factor), phosphatidylethanolamine (PE), alkylphosphatidylethanolamine (PE(O)), alkenylphosphatidylethanolamine (PE(P)), lysophosphatidylethanolamine (LPE), PI, lysophosphatidylinositol (LPI), phosphatidylserine (PS), DAG, TAG, dihydroceramide (dhCer), ceramide (Cer), monohexosylceramide (MHC), dihexosylceramide (DHC), trihexosylceramide (THC), monosialodihexosylganglioside (GM3) (Supplementary Table S1). The abbreviations shown above are only used when referring to individual lipid species, as in LPC (22:6), which defines a lysophosphatidylcholine with a fatty acid containing 22 carbons and six double bonds. For several lipids containing two fatty acid chains, the mass spectrometry-based measurements do not directly determine the constituent fatty acids but rather the sum of the number of carbons and the sum of the number of double bonds across both fatty acids.

**Supplementary Table S1. Analysis and quantitation of lipid species: Internal standards, parent ions, precursor ion scans**

**and neutral loss scans for tandem mass spectrometry.**

| **Lipid class or subclass** | **No. of species** | **Internal standard** | **Pmol^1^** | **Parent ion** | **Experiment^2^** |
| --- | --- | --- | --- | --- | --- |
| Dihydroceramide (dhCer) | 6 | dhCer 8:0 | 100 | [M+H]^+^ | PIS, 284.3 *m/z* |
| Ceramide (Cer) | 5 | Cer 17:0 | 100 | [M+H]^+^ | PIS, 264.3 *m/z* |
| Monohexocylceramide (MHC) | 6 | MHC 16:0 *d_3_* | 50 | [M+H]^+^ | PIS, 264.3 *m/z* |
| Dihexosylceramide (DHC) | 6 | DHC 16:0 *d_3_* | 50 | [M+H]^+^ | PIS, 264.3 *m/z* |
| Trihexosylceramide (THC) | 6 | THC 17:0 | 50 | [M+H]^+^ | PIS, 264.3 *m/z* |
| G_M3_ ganglioside (GM) | 6 | THC 17:0 | 50 | [M+H]^+^ | PIS, 264.3 *m/z* |
| Sphingomyelin (SM) | 21 | SM 12:0 | 200 | [M+H]^+^ | PIS, 184.1 *m/z* |
| Phosphatidylcholine (PC) | 51 | PC 13:0/13:0 | 100 | [M+H]^+^ | PIS, 184.1 *m/z* |
| Alkylphosphatidylcholine (PC(O)) | 19 | PC 13:0/13:0 | 100 | [M+H]^+^ | PIS, 184.1 *m/z* |
| Phosphatidylcholine plasmalogen (PC(P)) | 12 | PC 13:0/13:0 | 100 | [M+H]^+^ | PIS, 184.1 *m/z* |
| Lysophosphatidylcholine (LPC) | 21 | LPC 13:0 | 100 | [M+H]^+^ | PIS, 184.1 *m/z* |
| Lysoalkylphosphatidylcholine (LPC(O)) | 9 | LPC 13:0 | 100 | [M+H]^+^ | PIS, 285.2 *m/z* |
| Phosphatidylethanolamine (PE) | 19 | PE 17:0/17:0 | 100 | [M+H]^+^ | NL, 141 Da |
| Alkylphosphatidylethanolamine (PE(O)) | 11 | PE 17:0/17:0 | 100 | [M+H]^+^ | NL, 141 Da |
| Phosphatidylethanolamine plasmalogen (PE(P)) | 10 | PE 17:0/17:0 | 100 | [M+H]^+^ | NL, 141 Da |
| Lysophosphatidylethanolamine (LPE) | 6 | PE 14:0/0:0 | 100 | [M+H]^+^ | NL, 141 Da |
| Phosphatidylinositol (PI) | 16 | PE 17:0/17:0 | 100 | [M+NH_4_]^+^ | PIS, 184.1 *m/z* |
| Lysophosphatidylinositol (LPI) | 4 | PE 14:0/0:0 | 100 | [M+NH_4_]^+^ | NL, 277 Da |
| Phosphatidylserine (PS) | 6 | PS 17:0/17:0 | 100 | [M+H]^+^ | NL, 185 Da |
| Cholesteryl ester (CE) | 23 | CE 18:0 *d_6_* | 1000 | [M+NH_4_]^+^ | PIS, 369.3 *m/z* |
| Free cholesterol (COH) | 1 | COH *d_7_* | 1000 | [M+NH_4_]^+^ | PIS, 369.3 *m/z* |
| Diacylglycerol (DAG) | 19 | DG 15:0/15:0 | 200 | [M+NH_4_]^+^ | NL, fatty acid |
| Triacylglycerol (TAG) | 43 | TG 17:0/17:0/17:0 | 100 | [M+NH_4_]^+^ | NL, fatty acid |
| **Total lipid species** | **308** |  |  |  |  |

^1^ Amount of internal standard per sample. ^2^ PIS = precursor ion scan, NL = neutral loss scan.

Accordingly, we denote these species as the combined length and number of double bonds, eg. PC(36:4). The relative amounts of each molecular lipid species were calculated by expressing the peak area of each species relative to the peak area of the corresponding stable isotope or non-physiological internal standard as described previously (1,2,4,8). In targeted lipidomics, use of a stable isotope-labelled internal standard for each lipid species under investigation is not feasible. This approach does not provide absolute quantification but rather a close approximation of the actual concentration. Nonetheless, the precision of these measures is highly satisfactory (CV range 5-15%) and so the fold change (or % change resulting from treatment) is accurate. A correction factor of 10 was applied to the alkenylphosphatidylethanolamine species to account for the lower signal response of these lipid species relative to the PE(17:0/17:0) internal standard. Background measurements obtained from blank samples were then subtracted from all other samples in each run, including quality control samples. Concentrations of total lipid classes were calculated from the sum of the individual molecular lipid species within each class. To gain insight into the composition of the surface lipid mosaic of HDL particles, lipidomic data were normalised to molar content of the major surface lipid, PC; for insight into overall structural organisation, concentrations of lipid classes were normalised to the major HDL apolipoprotein, apoAI. It is relevant that (i) molar PC content per apoAI diminished markedly from light to dense HDL (see below), (ii) variation in the molar content of apoAI occurs across HDL subpopulations (5), and further that statin-mediated redistribution of apoAI between HDL particle subpopulations was observed post-statin (6).

Finally, lipidomic analyses by LC-MS do not permit precise determination of absolute lipid concentrations in either plasma or lipoprotein subclasses, and therefore cannot be compared directly with lipid measurements using clinical assays, as exemplified by data in Figure 1 and Supplementary Table S2 (1,2,4,8). However, LC-MS lipid quantitation allows comparison of data within a single data set obtained with the same technology, which extends to lipid extraction, LC-MS resolution of molecularly defined lipid classes and their constituent species, and detection and quantitation of these lipids using a suitable standard(s). When comparing absolute lipid class levels in HDL determined with clinical spectrophotometric assays against those in isolated HDL subclasses, multiple factors may contribute to variance in absolute values. These involve: i) lipoprotein losses during ultracentrifugal isolation and ii) the fact that clinical assays regroup lipid classes that are either completely or incompletely quantitated individually by MS; for example, both COH and CE are estimated together in HDL-C assays, but quantitated separately by LC-MS. Similarly, phospholipids are separated into up to 18 subclasses by LC-MS, while typically only those containing choline are measured clinically. Equally, total and partial glycerides are measured together on the basis of their glycerol content by clinical assays, but are quantitated as DAG and TAG by LC-MS.

**Statistical analyses of plasma and lipid parameters.** The effect of pitavastatin calcium treatment on each plasma parameter was determined by comparison of baseline values (D0) with corresponding values at D180 by the Student’s paired t-test corrected for multiple comparisons by the method of Benjamini-Hochberg. Data are expressed as means ± SEM or means % differences between groups for normally distributed variables and as median (minimum-maximum) for asymmetrically distributed parameters; distribution normality was assessed using the Kolmogorov-Smirnov test. P values were determined with the paired t-test analysis for values displaying a Gaussian distribution and with the non-parametric Wilcoxon test for those that were asymmetrically distributed. Given the markedly similar phenotype of our subjects on the one hand (1-4), and of the consistency of their response to statin treatment on the other (see figure 1 in ref 3)(2), inter-individual variability was attenuated, typically leading to a symmetrical distribution for most of the parameters determined. Correlation analyses of lipid parameters in our database were performed with the Pearson and Spearman tests as a function of the distribution of component values for a given parameter. For comparison of control subjects with HTG patients at baseline and at D180, the non-paired t-test was

performed. The determination of p for trend was made by repeated measures one-way ANOVA with a post-test for linear trend using GraphPadPrism® 4 software (GraphPad Software Inc, version 4.03); for analyses of molecular lipid species, GraphPad prism versions 8.0 and 8.1 were used.

**Supplementary Table S2: Weight % chemical composition and CE/TG ratio of HDL subclasses in hypertriglyceridemic subjects at baseline (D0) and the effect of pitavastatin calcium (4mg/day) treatment for 180 days (D180). Data extracted from Table 3 in ref 3.**

| Component | Table | HDL2b | HDL2a | HDL3a | HDL3b | HDL3c |
| --- | --- | --- | --- | --- | --- | --- |
| TAG | D0 | 14.7 ± 2.5 | 8.3 ± 1.0 | 6.4 ± 0.7 | 7.9 ± 1.2 | 9.0 ± 1.5 |
|  | D180 | **8.5 ± 1.2**** | **5.0 ± 0.4**** | **4.1 ± 0.4**** | **4.0 ± 0.4**** | **4.6 ± 0.7**** |
| CE | D0 | 24.0 ± 0.7 | 18.5 ± 0.8 | 18.6 ± 0.9 | 16.8 ± 0.9 | 14.4 ± 0.9 |
|  | D180 | 27.0 ± 1.8 | **20.7 ± 0.9***** | **20.5 ± 0.9*** | **18.3 ± 0.9**** | **16.1 ± 1.0*** |
| FC | D0 | 4.7 ± 0.2 | 2.7 ± 0.1 | 2.1 ± 0.1 | 1.8 ± 0.1 | 1.5 ± 0.3 |
|  | D180 | **5.1 ± 0.2*** | **3.0 ± 0.1**** | 2.2 ± 0.1 | 1.8 ± 0.1 | 1.1 ± 0.2 |
| PL | D0 | 25.7 ± 1.0 | 29.7 ± 0.7 | 27.6 ± 0.7 | 22.8 ± 0.6 | 16.5 ± 0.7 |
|  | D180 | 27.6 ± 0.9 | 30.4 ± 0.7 | 28.4 ± 0.6 | **24.7 ± 0.7**** | **18.5 ± 1.0**** |
| Total Protein | D0 | 30.8 ± 2.4 | 40.9 ± 1.6 | 45.4 ± 1.4 | 50.7 ± 2.1 | 58.6 ± 2.5 |
|  | D180 | 31.7 ± 2.6 | 41.0 ± 1.5 | 44.8 ± 1.4 | 51.1 ± 1.6 | 59.7 ± 2.3 |
| CE/TAG ratio | D0 | 2.2 ± 0.4 | 2.6 ± 0.3 | 3.4 ± 0.5 | 2.6 ± 0.4 | 2.1 ± 0.3 |
|  | D180 | 3.7 ± 0.4** | 4.5 ± 0.5*** | 5.6 ± 0.7* | 5.1 ± 0.6*** | 4.6 ± 0.9** |

Values are expressed as means ± SEM (n=12) (6). P values were determined by paired t-test analysis for values displaying Gaussian distribution and by the Wilcoxon non-parametric test for values that were non-Gaussian. ***p<0.001, **0.001<p<0.01 and *0.01<p<0.05 for D180 vs D0. Details of assay methods are provided in Supplemental Data and in ref 3. Total lipoprotein mass (calculated as the sum of TG, CE, FC, PL and total protein) was determined as described earlier (3,5,7). Molecular weights for HDL subclasses were reported earlier in normolipidemic subjects (7). Hydrated density ranges: HDL2b = 1.063-1.091 g/mL, HDL2a = 1.091-1.110 g/mL, HDL3a = 1.110-1.133 g/mL, HDL3b = 1.133-1.156 g/mL and HDL3c = 1.156-1.179 g/mL. Data extracted from ref 3 (n=12).

**RESULTS:**

**Pitavastatin-mediated remodeling of LpAI and LpAI:AII.** Both LpAI and LpAI:AII particles were primarily distributed in HDL2a and 3a at baseline, representing particles of intermediate size (3). Upon statin treatment, LpAI content in HDL3a was significantly reduced concomitantly with elevation in LpAI:AII in HDL2a and 3a as previously reported (3).

**Quantitative lipidomic profiles in HDL subclasses from hypertriglyceridemic subjects at baseline (D0) and post-statin (D180).** The intermediate HDL2a and 3a subclasses were the primary transporters (≈ 50-70% of total) of several neutral and polar lipid classes at baseline, including CE, glycerolipids, sphingomyelins, phosphatidylcholines, plasmalogens, PE, PI, PC(O), PE(O), and lysophospholipids (Suppl Figure S2). Concentrations of all lipid classes differed significantly among HDL subclasses at D0 (Suppl Figure S2). HDL2b, 2a and 3a were together the main transporters of free cholesterol and minor lipids (<1µmol/mL) including LPE, LPI, Cer, dhCer, and ceramide-related sphingolipids (MHC, DHC, THC and GM3). The cargo of TAG was some 3-fold greater than that of DAG in all HDL subclasses (Suppl Figure S2C, D). Bioactive LPC predominated (range ≈ 1-3 µmol/mL plasma) among lysophospholipids in HDL subclasses, with lower levels of LPE (range ≈ 0.1-0.35 µmol/mL), LPI (range 0.03-0.13 µmol/mL) and LPC(O) (≈ 0.01-0.33 µmol/mL) (Suppl Figure S2I, N, P, J). Plasma LpPLA2 activity was negatively correlated with the total lysolipid content of HDL2a (0.01<p<0.05) and equally with total LPC content (0.01<p<0.05) at baseline; similar correlations were observed in HDL3c for LPC and total lysolipids (both 0.01<p<0.05). Statin treatment induced significant increment (≈10-20%) in two abundant lipids, free cholesterol and SM, in HDL2a and 3b (Suppl Figure S2A, E; S4A, E). By contrast, a trend to lower contents of both TAG and DAG was observed; these decrements (up to 30%) attained significance in HDL2a and 3a for TAG and in HDL2b, 3a and 3b for DAG, but did not impact the DAG/TAG ratio (Suppl Figures 2C, D and S4X). The ratio of CE/TAG concentration trended to increase from large, light HDL2b to small dense HDL3c (Suppl Figure S2W), consistent with chemical analysis (Supplementary Table S2). Trends to reduction were seen in the lysoalkylphospholipid cargo (LPC(O)) of HDL2b and 3a (up to 15% p< 0.05) (Suppl Figure S2J). Trends to increase in abundance of plasmalogens PC(P) and PE(P), (up to 15%), were observed across all HDL subclasses, attaining significance for PC(P) in HDL3b and for PE(P) in HDL2a, 3a and 3b respectively (Suppl Figure 2G, L) (4). Minor increments (up to ≈15%; p< 0.05) occurred for Cer abundance in both HDL2a and 3a, and for MHC, DHC, THC and GM3 in HDL2a (Suppl Figure 2S, T, U, V).

**Supplementary figure S2:**

Plasma concentrations of individual plasma lipid classes across HDL subclasses at baseline (D0), and the effect of pitavastatin calcium treatment (4mg/day) for 180 days (D180) in hypertriglyceridemic subjects, with statistical comparisons. Values are expressed as means ± SEM (n=12) in pmol of each lipid class / mL plasma. ***p<0.001, **0.001<p<0.01 and *0.01<p<0.05 vs between subclasses; p values were calculated by Bonferroni test as a post-test to a two-way repeated measure ANOVA test. Panels A to Y: **A** - COH: free cholesterol; **B** - CE: cholesteryl ester; **C** - TAG: triacylglycerol; **D** - DAG: diacylglycerol; **E** - SM: sphingomyelin; **F** - PC: phosphatidylcholine; **G** - PC(P): alkenylphosphatidylcholine (plasmalogen); **H** - PC(O): alkylphosphatidylcholine; **I** - LPC: lysophosphatidylcholine; **J** - LPC(O): lysoalkylphosphatidylcholine; **K** - PE: phosphatidylethanolamine; **L** - PE(P): alkenylphosphatidylethanolamine (plasmalogen); **M** - PE(O): alkylphosphatidylethanolamine; **N** - LPE: lysophosphatidylethanolamine; **O** - PI: phosphatidylinositol; **P** - LPI: lysophosphatidylinositol; **Q** - Cer: ceramide; **R** - dhCer: dihydroceramide; **S** - MHC: monohexosylceramide; **T** -. DHC: dihexosylceramide; **U** – THC: trihexosylceramide; **V** – GM3: monosialodihexosylganglioside; **W** – CE/TAG ratio; **X** – DAG/TAG ratio; **Y** – SM/PC ratio.

**Supplementary Figure S2.**

**Supplementary figure S2.**

Supplementary Figure S2 (cont)

**Supplementary Figure S3.** Effect of pitavastatin calcium treatment (4mg/day) for 180 days (D180) on molar ratios of lipids expressed relative to moles apoAI at baseline (D0) in HDL2b, HDL2a, HDL3a, HDL3b and HDL3c subclasses in hypertriglyceridemic subjects Values are expressed as means ± SEM (n=12). ***p<0.001; **0.001<p<0.01 and *0.01<p<0.05 vs D0. **Panels A to V:** **A** - COH: free cholesterol; **B** - CE: cholesteryl ester; **C** - TAG: triacylglycerol; **D** - DAG: diacylglycerol; **E** - SM: sphingomyelin; **F** - PC: phosphatidylcholine; **G** - PC(P): alkenylphosphatidylcholine (plasmalogen); **H** - PC(O): alkylphosphatidylcholine; **I** - LPC: lysophosphatidylcholine; **J** - LPC(O): lysoalkylphosphatidylcholine; **K** - PE: phosphatidylethanolamine; **L** - PE(P): alkenylphosphatidylethanolamine (plasmalogen); **M** - PE(O): alkylphosphatidylethanolamine; **N** - LPE: lysophosphatidylethanolamine; **O** - PI: phosphatidylinositol; **P** - LPI: lysophosphatidylinositol; **Q** - Cer: ceramide; **R** - dhCer: dihydroceramide; **S** - MHC: monohexosylceramide; **T** -. DHC: dihexosylceramide; **U** – THC: trihexosylceramide; **V** – GM3: monosialodihexosylganglioside.

**Supplementary Figure S3.**

**Proteomic profiles in HDL subclasses from HTG subjects**

Proteomic analysis of HDL subpopulations isolated by isopycnic density gradient ultracentrifugation (IDGUC) revealed five distinct abundance patterns across subclasses (Supplementary Figure S4); importantly, such patterns were entirely consistent with the distribution of common HDL-associated proteins in fractions separated on the basis of size by gel filtration chromatography (GFC) (9). These findings indicate that redistribution of HDL-associated proteins does not occur to a significant degree in HDL subclasses isolated by IDGUC when GFC is used as the reference fractionation method; moreover, more than 95% of major HDL proteins were recovered in IDGUC fractions of d<1.21 g/mL (3,5,9).

**Supplementary Figure S4. Effect of pitavastatin treatment on proteomic profiles of HDL subclasses.** Effect of pitavastatin calcium treatment (4mg/day) for 180 days (D180) on the distribution of HDL proteins across five HDL subclasses in hypertriglyceridemic subjects (n=12). HDL proteins were quantified using MaxQuant and LFQAnalyst as described in Methods. The data represent the LFQ intensities of each protein detected in each HDL subclass, with the horizontal bar showing the mean value. Black dots show data at baseline (D0) and red dots show data post-statin (D180). *p<0.05 using a paired t-test with correction for multiple comparisons.

**
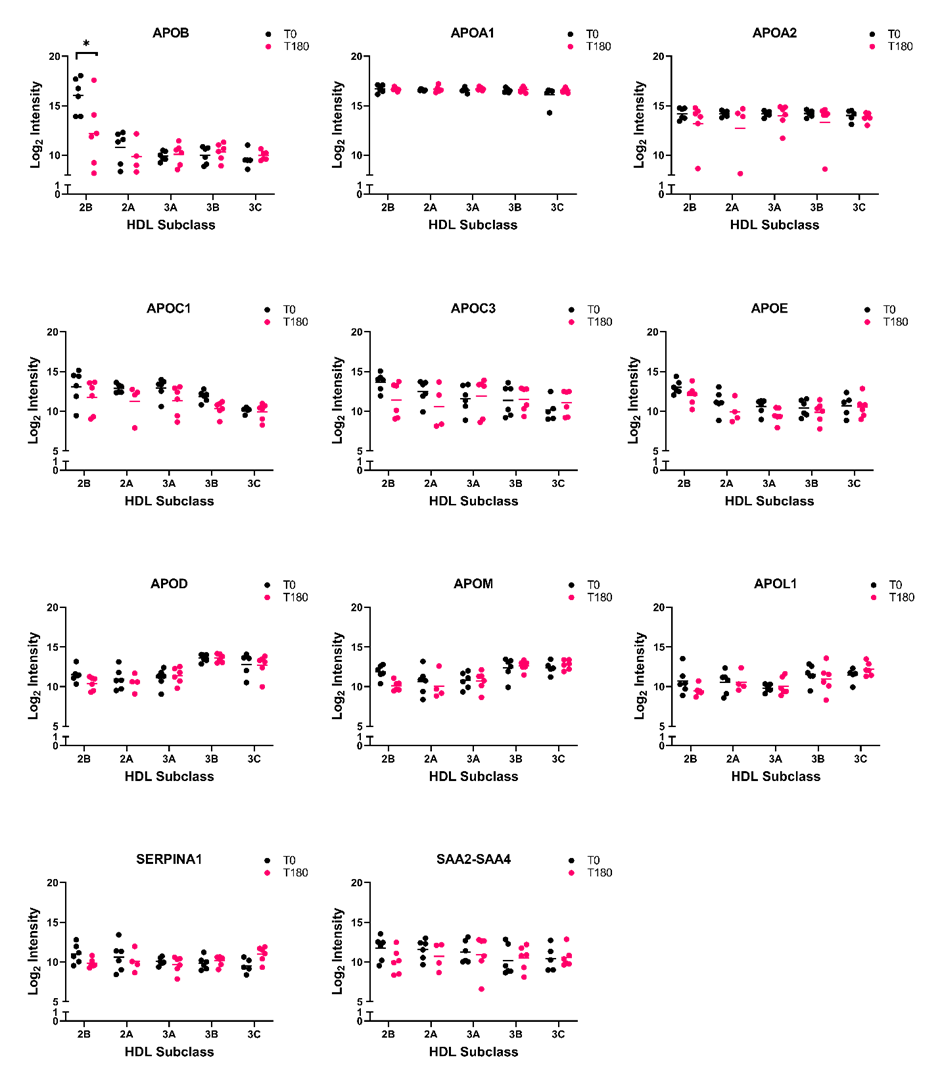
**

**Supplementary Figure S4**

**Supplementary Table S3: Effect of pitavastatin treatment (4mg/day; 180 days) on the composition of molecular species in the TAG lipidome, normalized to pmol PC, in HDL2b, HDL2a, HDL3a, HDL3b and HDL3c from hypertriglyceridemic subjects.**

| TAG species  (fmol/pmol PC) | HDL2b | | | |  | HDL2a | | | |
| --- | --- | --- | --- | --- | --- | --- | --- | --- | --- |
|  | D0 | D180 | % change^a^ | p-value |  | D0 | D180 | % change^a^ | p-value |
| TAG(16:0-16:0-18:1) | 9.55 ± 0.61 (11%) | 7.06 ± 0.69 | -26 | **0.0157** |  | 7.80 ± 0.49 (11%) | 5.63 ± 0.64 | -28 | **0.0123** |
| TAG(16:0-16:1-18:1) | 9.11 ± 0.57 (10%) | 6.62 ± 0.68 | -27 | **0.0191** |  | 7.87 ± 0.50 (11%) | 5.46 ± 0.52 | -31 | **0.0045** |
| TAG(16:0-18:1-18:1) | 17.72 ± 0.84 (20%) | 14.50 ± 0.84 | -18 | **0.0118** |  | 15.08 ± 0.83 (21%) | 11.56 ± 0.78 | -23 | **0.0008** |
| TAG(16:0-18:1-18:2) | 8.75 ± 0.77 (10%) | 6.15 ± 0.43 | -30 | **0.0052** |  | 7.40 ± 0.58 (10%) | 4.91 ± 0.48 | -34 | **<0.0001** |

| TAG species  (fmol/pmol PC) | HDL3a | | | |  | HDL3b | | | |
| --- | --- | --- | --- | --- | --- | --- | --- | --- | --- |
|  | D0 | D180 | % change^a^ | p-value |  | D0 | D180 | % change^a^ | p-value |
| TAG(16:0-16:0-18:1) | 7.28 ± 0.60 (11%) | 5.22 ± 0.60 | -28 | **0.0062** |  | 7.37 ± 0.54 (11%) | 5.91 ± 0.87 | -20 | 0.1577 |
| TAG(16:0-16:1-18:1) | 7.29 ± 0.49 (11%) | 5.11 ± 0.55 | -30 | **0.0028** |  | 7.38 ± 0.54 (11%) | 5.69 ± 0.75 | -23 | 0.0986 |
| TAG(16:0-18:1-18:1) | 13.97 ± 0.48 (21%) | 11.08 ± 0.75 | -21 | **0.0009** |  | 14.28 ± 0.73 (21%) | 11.95 ± 1.09 | -16 | 0.1175 |
| TAG(16:0-18:1-18:2) | 6.74 ± 0.44 (10%) | 4.77 ± 0.45 | -29 | **<0.0001** |  | 6.93 ± 0.58 (10%) | 5.32 ± 0.66 | -23 | **0.0244** |

| TAG species  (fmol/pmol PC) | HDL3c | | | |
| --- | --- | --- | --- | --- |
|  | D0 | D180 | % change^a^ | p-value |
| TAG(16:0-16:0-18:1) | 14.5 ± 1.81 (10%) | 9.26 ± 1.48 | -36 | **0.0182** |
| TAG(16:0-16:1-18:1) | 15.51 ± 2.19 (10%) | 9.35 ± 1.43 | -40 | **0.0108** |
| TAG(16:0-18:1-18:1) | 25.78 ± 3.03 (17%) | 17.27 ± 1.80 | -33 | **0.0237** |
| TAG(16:0-18:1-18:2) | 14.82 ± 2.31 (10%) | 8.62 ± 1.08 | -42 | **0.0162** |

Only species which represent at least 5% of the total class are presented. Values are expressed as mean ± SEM (n=12). In brackets, percent of species to total class. P values were determined with the paired t-test analysis for values displaying a Gaussian distribution and with the non- parametric Wilcoxon test for values that did not display a Gaussian distribution. All values highlighted in bold are statistically significant at the level of ≤0.05. ^a^Percent change from baseline (D0) after pitavastatin (4mg/day) treatment for 180 days (D180).

**References**

1. Meikle PJ, Wong G, Tan R, Giral P, Robillard P, Orsoni A, et al. Statin action favors normalization of the plasma lipidome in the atherogenic mixed dyslipidemia of MetS: potential relevance to statin-associated dysglycemia. *J Lipid Res*. 2015; **56** :2381-92.

2. Chapman MJ, Orsoni A, Tan R, Mellett NA, Nguyen A, Robillard P, et al. LDL subclass lipidomics in atherogenic dyslipidemia: effect of statin therapy on bioactive lipids and dense LDL. *J Lipid Res*. 2020; **61** :911-932.

3. Chapman MJ, Orsoni A, Robillard P, Therond P, Giral P. Duality of statin action on lipoprotein subpopulations in the mixed dyslipidemia of metabolic syndrome: Quantity vs quality over time and implication of CETP. *J Clin Lipidol.* 2018; **12**: 784-800.e4.

4. Orsoni A, Thérond P, Tan R, Giral P, Robillard P, Kontush A, et al. Statin action enriches HDL3 in polyunsaturated phospholipids and plasmalogens and reduces LDL-derived phospholipid hydroperoxides in atherogenic mixed dyslipidemia. *J Lipid Res.* 2016 ;**57** :2073-2087.

5. Chapman MJ, Goldstein S, Lagrange D, Laplaud PM. A density gradient ultracentrifugal procedure for the isolation of the major lipoprotein classes from human serum. *J Lipid Res*. 1981; **22**: 339–358.

6. Davidson WS, Silva RA, Chantepie S, Lagor WR, Chapman MJ, Kontush A. Proteomic analysis of defined HDL subpopulations reveals particle-specific protein clusters: relevance to antioxidative function. *Arterioscler Thromb Vasc Biol* 2009; **29**:870–876.

7. Kontush A, Therond P, Zerrad A, Couturier M, Négre-Salvayre A, de Souza JA, et al. Preferential sphingosine-1-phosphate enrichment and sphingomyelin depletion are key features of small dense HDL3 particles: relevance to antiapoptotic and antioxidative activities. *Arterioscler Thromb Vasc Biol.* 2007; 27:1843-9.

8. Meikle PJ, Wong G, Barlow CK, Weir JM, Greeve MA, MacIntosh GL, et al. Plasma lipid profiling shows similar associations with prediabetes and type 2 diabetes. *PLoS One.* 2013; 8: e74341.

9. Gordon SM, Deng J, Lu LJ, Davidson WS. Proteomic characterization of human plasma high density lipoprotein fractionated by gel filtration chromatography. *J Proteome Res.* 2010; **9**:5239-49.
